# PDLIM4 drives gastric cancer malignant progression and cisplatin resistance by inhibiting HSP70 ubiquitination and degradation via competitive interaction with STUB1

**DOI:** 10.1186/s12951-025-03720-4

**Published:** 2025-10-11

**Authors:** Chao Zhu, Meng Chen, Linwei Fan, Yu Wang, Mengwei Liu, Guiyu Kang, Fang Yin, Hong Tang, Yun He, Sifan Zhang, Linda Zeng, Wei Liu, Kuai Yu, Aiping Le

**Affiliations:** 1 Department of Transfusion Medicine, Key Laboratory of Jiangxi Province for Transfusion Medicine, The First Affiliated Hospital, Jiangxi Medical College, Nanchang University, Nanchang, 330006 Jiangxi China; 2 Postdoctoral Research Station, The First Affiliated Hospital, Jiangxi Medical College, Nanchang University, Nanchang, 330006 People’s Republic of China; 3 Department of Hematology, The First Affiliated Hospital of Nanchang University, Nanchang, 330006 China

**Keywords:** PDLIM4, STUB1/HSP70, Ubiquitination, Lipid nanoparticles, Gastric cancer, DDP

## Abstract

Gastric cancer (GC) frequently shows malignant progression and resistance to chemotherapy due to complex molecular regulatory processes, leading to a poor prognosis. Our study elucidates that PDZ and LIM domain protein 4 (PDLIM4) are highly expressed in GC, thereby promoting the malignant progression and cisplatin (DDP) resistance of GC. Mechanistically, PDLIM4, via its C-terminal and intermediate regions, impedes the ubiquitination and proteasomal degradation of Heat Shock Protein 70 (HSP70) by competing with the STIP1 homology and U-box containing protein 1 (STUB1), subsequently activating the MAPK signaling pathway. In addition, we synthesized lipid nanoparticles (LNPs), including siPDLIM4 LNPs, DDP LNPs, and siPDLIM4/DDP LNPs. Experiments further indicated that siPDLIM4 LNPs and DDP LNPs have an anti-tumor property, while siPDLIM4/DDP LNPs exhibit the most significant anti-tumor efficacy. In summary, our research identifies PDLIM4 as a facilitator of malignant progression and DDP resistance in GC cells, targeting PDLIM4 not only inhibits the malignant progression of GC, but also provides an effective strategy to enhance DDP sensitivity in GC treatment, emphasizing its potential as a therapeutic target.

**Graphical Abstract:**

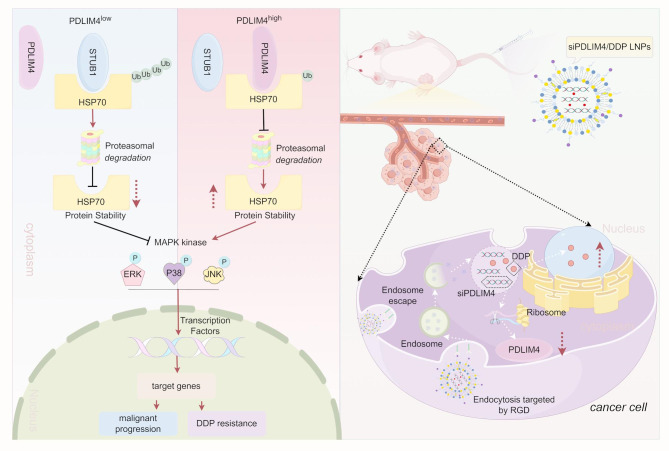

**Supplementary Information:**

The online version contains supplementary material available at 10.1186/s12951-025-03720-4.

## Introduction

The latest data from the International Agency for Research on Cancer (IARC) shows that gastric cancer (GC) is the fifth most prevalent form of cancer and the fifth leading cause of cancer-related mortality globally [1]. Nearly 1,000,000 new cases and over 650,000 deaths reported annually, GC remains a significant global health issue [2]. While the application of a chemotherapy drug-cisplatin's (DDP) effectively delayed the progression of GC, the climbing events of DDP resistance occur and even led to the failure of treatment. Various molecular mechanisms are involved in this resistance. ATF3 is considered a potential enhancer of GC cells' sensitivity to DDP by causing ferroptosis [3]. Additionally, Rho GDP dissociation inhibitor 2 (RhoGDI2) enhances Bcl-2 expression, conferring DDP-induced apoptosis resistance in GC cells [4]. The malignancy of GC and its resistance to chemotherapy are often attributed to its intricate molecular mechanisms [5–7], which contribute to the poor prognosis found in patients. As a result, finding novel biomolecules is vital, as it might pave the way for potential GC treatments, emphasizing the significance of clarifying the molecular processes involved in GC progression.

PDZ and LIM domain protein 4 (PDLIM4) belongs to the PDLIM family, which also includes members such as PDLIM1, 2, 3, 5 and 7. PDLIM4 contains conserved PDZ and LIM domains [8]. Findings suggest that PDLIM4 is implicated in the reorganization of the actin cytoskeleton and the movement of cells [9], positioning it as a key regulator of cytoskeletal dynamics and cancer metastasis. Moreover, the role of PDLIM4 is significant in the progression of different tumors, where it demonstrates unique tumor-suppressive properties by modulating the transcription factor STAT3 [10]. Additionally, higher methylation levels of PDLIM4 could act as a sensitive molecular marker for identifying prostate cancer [11]. In summary, the role of PDLIM4 in cancer is multifaceted, encompassing aspects of gene expression regulation and methylation status. Comprehensive investigations into the function and regulatory mechanisms of PDLIM4 might provide new understanding and possible targets for diagnosing and treating GC. Our bioinformatic analysis reveals a significant association between PDLIM4 expression and the clinical characteristics and outcomes in GC patients. However, the specific mechanisms underlying PDLIM4's role in GC remain to be elucidated. Our research indicates that PDLIM4 could be a possible therapeutic target for GC.

Heat Shock Protein 70 (HSP70) is distinguished by its highly conserved and functional diversity [12]. Structurally, HSP70 comprises two distinct domains: the N-terminal ATPase domain, which regulates energy cycling, and the C-terminal domain, which is responsible for substrate binding [13, 14]. HSP70 plays a crucial role in the regulation of protein metabolism, stress responses, and developmental processes [15], and has been implicated in tumor progression [16, 17]. The existing literature has established a correlation between HSP70 and the progression of GC [18, 19]. The STIP1 homology and U-box containing protein 1 (STUB1), which is functionally related to HSP70 and located at locus 16q13.3, encodes both a U-box domain and a tetratricopeptide repeat (TPR) domain [20]. STUB1 serves dual functions, acting as an E3 ubiquitin ligase while also assisting as a molecular chaperone [21]. The involvement of STUB1 in tumor development and progression has garnered considerable interest. STUB1 facilitates the ubiquitination and subsequent degradation of GPX4, thereby inhibiting tumor growth [22]. There is literature proving that STUB1 interacts with the C-terminus of HSP70 to regulate its stability [23]. Previous research has demonstrated that STUB1 facilitates the degradation of Foxp3 in regulatory T cells via the HSP70-dependent pathway, consequently diminishing its immunosuppressive function [24]. Furthermore, the STUB1/HSP70 complex is pivotal in modulating protein stability [25]. Our findings further reveal that STUB1 interacts with the C-terminus of HSP70, enhancing the ubiquitination and proteasomal degradation of HSP70. Nonetheless, the upstream regulatory mechanisms governing the STUB1/HSP70 signaling pathway remain elusive. To address this issue, our team conducted experiments, revealing that PDLIM4 can inhibit the interaction between STUB1 and HSP70, thereby impacting HSP70 stability.

Lipid nanoparticles (LNPs) are characterized by their ultra-small size typically ranging from 1 to 1000 nm, which are regarded as a promising vehicle for the clinical delivery of nucleic acid drugs[26]. The simultaneous encapsulation of siRNA and chemotherapeutic agents within LNPs presents a synergistic therapeutic approach, wherein siRNA effectively silences oncogenes, while chemotherapeutic agents exert direct cytotoxic effects on tumor cells [27–29]. Furthermore, LNPs are associated with low toxicity and enhanced tumor accumulation [30, 31]. The structural similarity between liposomes and cell membranes confers upon them enhanced capabilities for drug encapsulation and stability, thereby effectively safeguarding the drugs from degradation within the internal environment [32]. Numerous studies indicate that LNPs for nucleic acid drug delivery hold significant promise as a treatment approach for cancer. Therefore, our research team has developed a LNP system specifically designed for the targeted delivery of siPDLIM4 (siPDLIM4 LNPs), DDP (DDP LNPs) or siPDLIM4 along with DDP (siPDLIM4/DDP LNPs) to GC.

This study investigates the role of PDLIM4 in GC. Our initial findings reveal that the deletion of PDLIM4 can suppress malignant progression and boost the sensitivity of GC cells towards DDP. Mechanistically, PDLIM4 interacts with the C-terminal of HSP70 through its own C-terminal and intermediate domains. This interaction inhibits the binding of STUB1 to HSP70, thereby preventing STUB1-mediated ubiquitination and proteasomal degradation of HSP70. As a result, elevated levels of HSP70 activate the MAPK signaling pathway. Our results indicate that both siPDLIM4 LNPs and DDP LNPs effectively inhibit tumor growth, with siPDLIM4/DDP LNPs exhibiting the best anti-tumor effect. These findings might improve our comprehension of the molecular processes in GC progression and aid in creating new treatments.

## Methods

The detailed methods of Cell Lines and Cell Culture, Quantitative Real-Time PCR (RT-qPCR), Cell proliferation, migration assay, Cell survival assay, Cell apoptosis analysis, Identification of Prognosis-Related Genes in Gastric Cancer, Drug Sensitivity Analysis, Stability assay, Cellular Uptake, In vitro Transfection Efficiency are described in Supplementary Experimental Procedures.

### Tissue specimens

A total of 40 pairs of fresh GC tissues and adjacent normal tissues were obtained under the supervision of gastroenterologists. These samples were maintained at − 80 °C to be used in subsequent RT-qPCR and Western blotting analyses, or fixed in 10% formalin and embedded in paraffin for immunohistochemistry (IHC). Additionally, The Pathology Department at the First Affiliated Hospital of Nanchang University provided 210 paraffin-embedded GC tissue samples. All the samples originated from patients diagnosed with GC who underwent surgical procedures between January 2017 and January 2019, none of whom had received any prior treatment. The Ethics Committee of the First Affiliated Hospital of Nanchang University approved the study, and all participants provided informed consent (Approval Number: (2025) CDYFYYLK (04–030)).

### siRNA, shRNA, plasmid construction, and cell transfection

General Biol (Anhui) Co., Ltd (Anhui, China) synthesized the siRNA and shRNA primers, with their sequences listed in Table S1. A segment of SFB, whose sequence is also listed in Table S1, was fused to the N-terminus of PDLIM4 to facilitate binding with S-Protein Agarose and Flag antibody. This construct was inserted into the PCDH-CMV-MCS-EF1-copGFP-T2A-Puro vector, resulting in the SFB-PDLIM4 construct. Variants of SFB-PDLIM4, including SFB-PDLIM4 (330AA), SFB-PDLIM4 (1-88AA), SFB-PDLIM4 (1-260AA), SFB-PDLIM4 (80-260AA), SFB-PDLIM4 (80-330AA), and SFB-PDLIM4 (250-330AA) were procured from PPL (Public Protein/Plasmid Library, China). The HSP70 variants, HSP70-ΔN, HSP70-ΔC, and HSP70-ΔABD were procured from LuoRui Biotech (Jiangsu, China). For siRNA and plasmid transfections, Lipo3000 (Thermo Fisher Scientific, USA) and jet PRIME® (Polyplus, France) were employed, respectively. To generate stable cell lines with either knockdown or overexpression of PDLIM4, we utilized psPAX2, pMD2.G, and the target plasmid for lentiviral packaging. The resulting viral supernatant was then used to infect MKN45 and HGC27 cells.

### Western blotting

Total Proteins were split by SDS-PAGE, afterwards shifted to 0.45 μm nitrocellulose membranes (Millipore, USA). The membranes were incubated overnight with the primary antibody following 2 h block using 5% milk. Membranes were exposed to a secondary antibody the next day. Image acquisition was conducted using the UVP/ChemStudio Imaging System (Analytik Jena AG, Germany). The antibodies used included PDLIM4 (Abcam, Cat# ab251701), HSP70 (Proteintech, Cat# 10995–1-AP), STUB1 (Abmart, Cat# PHM9480), β-actin (ABclonal, Cat# AC026), GAPDH (Proteintech, Cat# 81640–5-RR), MYC (Proteintech, Cat# 16286–1-AP), Ub (Proteintech, Cat# 10201– 2-AP), HA (ABclonal, Cat# AE008), HA (Proteintech, Cat# 51064–2-AP), DYKDDDDK (Proteintech, Cat# 20543–1-AP), DYKDDDDK (Proteintech, Cat# 66008–4-Ig), P21 (ABclonal, Cat# A19094), P27 (ABclonal, Cat# A19095), bcl2 (Servicebio, Cat# GB154380), Bax (Servicebio, Cat# GB11690), E-cadherin (Proteintech, Cat# 20874–1-AP), MMP2 (Wanleibio, Cat# WL03224), MMP3 (Proteintech, Cat# 66338–1-Ig), MMP9 (Huabio, Cat# ET174-69), Vimentin (Huabio, Cat# ET1610-39), ERK (Proteintech, Cat# 11257–1-AP), P38 (Proteintech, Cat# 66234–1-Ig), JNK (Proteintech, Cat# 66210–1-Ig), p-ERK (Cohesion, Cat# CQA8093), p-P38 (Proteintech, Cat# 28796–1-AP), p-JNK1/2/3 (Cohesion, Cat# CQA8172), Integrin Alpha V + Beta3 (absin, Cat# abs122318), Integrin Alpha V + Beta3 (LMAI, Cat# LM-1310R), Rabbit IgG (Proteintech, Cat# 30000–0-AP) and Mouse IgG (Proteintech, Cat# B900620).

### Immunohistochemistry (IHC)

The paraffin-embedded sections underwent deparaffinization and rehydration, antigen retrieval was performed with EDTA (pH 9.0). Subsequently, they were immersed in a 3% hydrogen peroxide solution for 15 min. Sections were incubated overnight at 4 °C with primary antibodies after 2 h block with 5% milk, including PDLIM4 (Abmart, Cat# PC6093M), HSP70 (Proteintech, Cat# 10995–1-AP), and Ki67 (Huabio, Cat# HA721115). The next day, the sections were treated with a secondary antibody for 1 h, then stained with DAB and counterstained with hematoxylin. A scoring system was employed to evaluate the degree of staining, assessing the proportion of positive cells using the following scale: 0 (0%), 1 (1–24% positive cells), 2 (25–49% positive cells), 3 (50–74% positive cells), and 4 (≥ 75% positive cells). The intensity of staining was rated as 0 for negative, 1 for weak, 2 for moderate, and 3 for strong. The immune reactivity score was obtained by multiplying the degree score with the intensity score, with the median value used to differentiate between low and high expression.

### Immunofluorescence (IF)

MKN45 and HGC27 cells, transfected with SFB-PDLIM4 and HSP70-HA, were fixed using 4% paraformaldehyde for a duration of 30 min. Following this, 0.2% Triton X-100 was used to permeabilize the cells for 15 min. Following permeabilization, the cells were blocked with 5% goat serum for 1 h. Primary antibodies were used to incubate the cells overnight at 4 °C, and the next day, secondary antibodies were applied for 1 h. Imaging was conducted utilizing a ZEISS confocal laser scanning microscope (Germany).

### Co-immunoprecipitation

The designated plasmids were used to transfect the cells, which were then lysed using IP lysis buffer (Servicebio, China) containing 50 × Cocktail (Servicebio, China). The cell lysates underwent incubation at 4 °C overnight for co-immunoprecipitation using specified antibody in conjunction with S-Protein Agarose (Millipore, USA) or Protein A/G Plus Sepharose 4FF Affinity Chromatography Resin (Sangon Biotech, China). The beads were washed with TBS buffer at least five times the next day. The final products were boiled in 2 × SDS-PAGE loading buffer (Solarbio, China) at 100 °C for 10 min. The immunoprecipitated samples were analyzed via western blotting.

### Proximity ligation assay (PLA)

The PLA was performed using a NaveniFlex Cell Red kit (Navinci, Sweden). On the first day, gastric cancer cells were seeded onto slides, and on the second day, they were fixed and permeabilized. After blocking non-specific binding with Naveni Block, the samples were incubated overnight with primary antibodies (rabbit anti-PDLIM4 and mouse anti-HSP70 (Proteintech, Cat#66183–1-Ig) or mouse anti-IgG). Navenibody were applied and incubated after 1 × TBST washing, with subsequent ligation and amplification steps. Finally, we cleaned the slides and performed nuclear staining using DAPI (Beyotime, China), and took photos using a ZEISS confocal laser scanning microscope (Germany).

### Mouse xenograft model

The animal research received approval from the Ethics Committee of the First Affiliated Hospital of Nanchang University (Ethics Number: CDYFY-IACUC-202309QR019). Hangzhou Ziyuan Laboratory Animal Technology Co., Ltd. supplied female BALB/c nude mice that were four weeks old. For the mouse xenograft tumor assay, nude mice received a subcutaneous injection of about 5 million cells, and the tumor volume was measured every three days. The calculated using the formula: volume = length × width^2 × 0.5. The mice were euthanized when the tumors grew to a size of 15 mm in diameter, and the tumors were either embedded in paraffin or kept at −80 °C.

### Synthesis of siPDLIM4 LNPs, DDP LNPs and siPDLIM4/DDP LNPs

Lipid nanoparticles (LNPs) were created by dissolving MC3, DSPC, DMG-PEG2000, cholesterol, and DSPE-PEG-RGD in ethanol with a molar ratio of 50:10:1.5:38.5:1. Subsequently, the lipid mixture and siRNA were combined at a nitrogen-to-phosphate (N/P) ratio of 6, which prepare LNPs encapsulating siPDLIM4 or/and DDP using a microfluidic cartridge.

### Characterization of lipid nanoparticles

The size and zeta potential of the nanoparticles were measured using the Zetasizer Nano ZS90 (Malvern Instruments, UK). Transmission electron microscopy (TEM, Japan) was utilized to analyze the nanoparticles' morphological characteristics. The encapsulation efficiency of siPDLIM4 was assessed using ultraviolet–visible spectroscopy (UV–Vis), while the encapsulation efficiency of DDP was assessed using inductively coupled plasma optical emission spectrometry (ICP-OES). Firstly, we used ICP-OES: Agilent 5110 to detect the Pt content, which is converted to 1 μg/ml Pt≈1.538 μg/ml DDP. We diluted the siPDIM4/DDP LNPs 50 times and measured the average concentration of Pt is 3.786 mg/l, so the Pt concentration in the siPDIM4/DDP LNPs is 189.3 mg/l, which is equivalent to DDP concentration of 291.16 mg/l. We synthesized the siPDIM4/DDP LNPs a total of 10 ml, therefore the content of DDP is 2.9116 mg. 5 mg DDP were used in our synthetic material, the encapsulation efficiency of DDP is 2.9116 mg/5 mg = 58.23%. The concentration of the siPDIM4/DDP LNPs is 3 mg/ml and the volume is 10 ml, therefore, the DDP loading capacity = encapsulated DDP mass/encapsulated DDP mass + carrier mass (2.9116 mg/2.9116 mg + 30 mg = 8.85%).

### Cisplatin release profile from LNPs

We conducted a drug release curve in PH6.0 PBS, which is a tumor-mimicking environments. We used the siPDIM4/DDP LNPs with a volume of 1 ml and a concentration of 3 mg/ml for drug release. Then we put it into a dialysis bag, the dialysis solution was 30 ml PBS buffer with pH 6.0. Later, we took out 1 ml dialysis solution at regular intervals and measured the content of Pt released by ICP-OES: Agilent 5110, and converted it to DDP content. The cumulative release = release DDP amount/total DDP amount.

### Pharmacokinetic assay and biodistribution of SIPDIM4/DDP LNPS in mice

Healthy female mice were assigned into 3 groups randomly (n = 3), with every group receiving a tail vein injection of either naked siPDLIM4, or siPDLIM4 LNPs, siPDLIM4/DDP LNPs, each at a dose of 1 nmol siRNA. The orbital vein was used to collect blood samples using heparinized tubes at predetermined intervals. The fluorescence intensity of CY5-labeled siPDLIM4 in the blood was calculated using a microplate reader to assess pharmacokinetics. For the biodistribution study, healthy female BALB/c nude mice with subcutaneous tumors were randomly divided into two groups (n = 16), with each group receiving an intravenous injection of either naked Cy5-siPDLIM4 or Cy5-siPDLIM4/DDP LNPs. After 1, 6, 24 h, the fluorescence intensity of CY5-siPDLIM4 in major organs and tumors was measured using the small animal CT/live imaging all-in-one machine (Milabs B.V.).

### Anti-tumor efficacy and toxicity of siPDLIM4 LNPs, DDP LNPs and siPDLIM4/DDP LNPs

In the mouse xenograft tumor assay, nude mice received subcutaneous injections of approximately 5 million MKN45 cells. Then, on the sixth day the mice of xenograft tumors were divided into five groups (n = 6) through random allocation. On the seventh day, the mice received injections via the tail vein (This injection method refers to relevant literatures [26, 29, 30, 33, 34]) with either PBS, LNPs, siPDLIM4 LNPs, DDP LNPs, or siPDLIM4/DDP LNPs with 1 nmol siRNA or/and DDP dosage of 2 mg/kg, administered every three days for a total of six cycles, which primarily informed by existing literatures [35–37]. On the 25th day, the mice were executed, and their major organs and tumors were harvested and preserved in 4% paraformaldehyde for IHC and HE staining. Servicebio (Wuhan, China) conducted an analysis of blood biochemical parameters such as alanine aminotransferase (ALT), aspartate aminotransferase (AST), urea, and creatinine (Crea).

### Data sources

The Cancer Genome Atlas (TCGA) database (http://cancergenome.nih.gov/) provided us with clinical information and RNA-seq transcriptomic data on GC. Additionally, four datasets—GSE66229, GSE122401, GSE179252, and GSE184336 were derived from the Gene Expression Omnibus (GEO) database (http://www.ncbi.nlm.nih.gov/geo/). The GDSC2 drug sensitivity data were accessed from the Genomics of Drug Sensitivity in Cancer (GDSC) database via GitHub (https://github.com/maese005/oncoPredict/tree/main/vignettes). Samples lacking complete clinical information were deemed ineligible and subsequently excluded from the study. Following the preprocessing of raw data, which encompassed sample deduplication, data normalization, probe annotation, gene filtering, and clinical grouping. To analyze differential gene expression between tumor and normal tissues, we used the Lima package in R.

### Statistical analysis

We used R and GraphPad Prism 9.0 for performing statistical analyses. Results are displayed as the mean ± standard deviation based on three different experiments. For differences between two groups, a t-test was utilized, whereas two-way or one-way ANOVA was employed for comparisons among multiple groups. Spearman's rank order correlation was used to perform the correlation analysis. A P-value below 0.05 was regarded as a sign of statistical significance.

## Results

### PDLIM4 is an independent prognostic factor for poor prognosis in GC patients

**Fig. 1:**
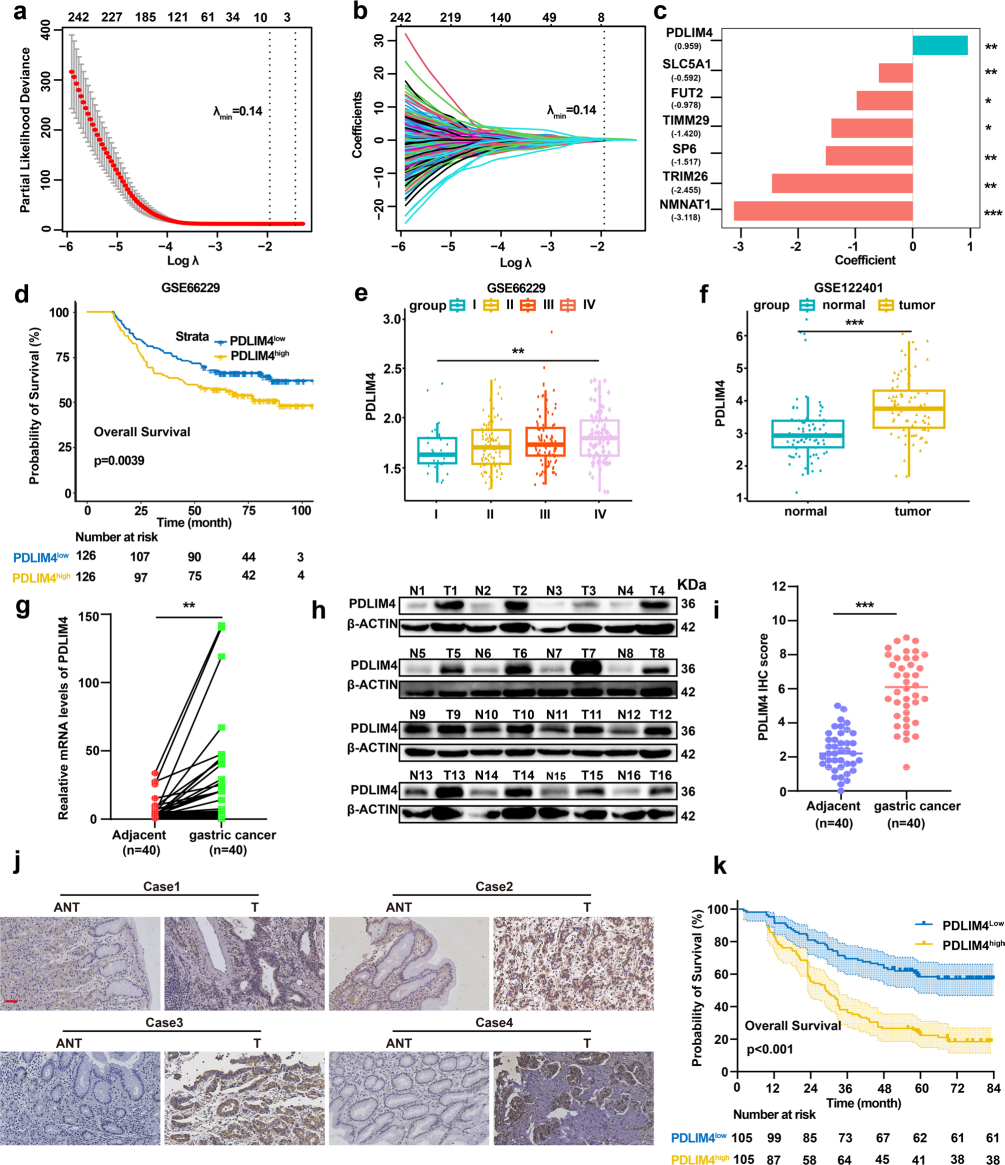
PDLIM4 is an independent prognostic factor for poor prognosis in GC patients.a-b A Lasso regression model was developed using the 551 genes from the GSE66229 dataset to determine the optimal parameter (λ). c Seven genes associated with prognosis were selected based on this optimal parameter (λ). d In the GSE66229 dataset, overall survival (OS) was stratified by high and low expression levels of PDLIM4. e Variations in PDLIM4 expression across different clinical stages of GC were observed in the GSE66229 dataset. f The GSE122401 dataset revealed differences in PDLIM4 expression between GC and normal tissues. g Quantitative analysis of PDLIM4 mRNA expression was performed on 40 pairs of GC tissues and their adjacent normal tissues. h Western blotting analysis was conducted to evaluate PDLIM4 protein levels in 16 pairs of GC tissues and adjacent normal tissues, with representative results displayed. i Immunohistochemistry (IHC) scoring was employed to assess PDLIM4 expression in 40 pairs of GC tissues and adjacent normal tissues. j Representative images of PDLIM4 IHC staining are provided for 4 pairs of GC tissues and adjacent normal tissues, scale bars: 50μm. k Patients were divided into two groups based on PDLIM4 expression and Kaplan Meier survival analysis was performed to examine the overall survival associated with PDLIM4 expression levels. Data were presented as means ± SD. *P<0.05; **P<0.01; ***P<0.001.

An intersection analysis of differentially expressed genes derived from univariate Cox and Kaplan–Meier (K-M) analyses within The Cancer Genome Atlas (TCGA) cohorts and the Gene Expression Omnibus (GEO) database (GSE66229) (comprising 551 genes identified seven genes suitable for prognostic modeling through Lasso regression (λmin = 0.14) (Fig. 1a, b; Table S2). Considering the scientific feasibility, technical challenges, and prospects of clinical translation, we opted to focus our study on oncogenes. The findings indicated that PDLIM4 (coefficient = 0.959, P < 0.01) serves as a significant prognostic factor (Fig. 1c, Table S3). Utilizing the median gene expression value of PDLIM4 from the GSE66229 dataset and TCGA cohort, patients were divided into PDLIM4 low (PDLIM4^low^) and PDLIM4 high (PDLIM4^high^) groups. Kaplan–Meier analysis demonstrated that in the GSE66229 cohort, the PDLIM4^high^ group emerged remarkably poorer OS in contrast with the PDLIM4^low^ group (Fig. 1d). Similarly, in the TCGA cohort, the PDLIM4^high^ group experienced worse outcomes in OS, Progression-Free Interval (PFI) and Disease-Specific Survival (DSS) relative to the PDLIM4^low^ group (Figure S1a-c). Furthermore, elevated PDLIM4 expression was associated with progressive tumor stage in the GSE66229 database (Fig. 1e) and PDLIM4 was obviously elevated in tumor tissues versus adjacent normal tissues in GSE122401, GSE179252, and GSE184336 datasets (Fig. 1f, Figure S1d-e). According to bioinformatics analysis, PDLIM4 might serve as a prognostic indicator in GC. Subsequently, to confirm the clinical relevance of PDLIM4 expression in GC, we employed the mRNA levels of PDLIM4 in 40 fresh pairs of GC tissues with RT-qPCR. In GC tissues, the mRNA expression level of PDLIM4 was notably increased (Fig. 1g). In addition, the protein expression of PDLIM4 was found to be markedly higher in GC tissues than in adjacent normal tissues, as confirmed by both western blotting and immunohistochemistry (Fig. 1h-j). Then, we categorized the patients into PDLIM4^high^ group (n = 105) and PDLIM4^low^ group (n = 105) based on the median IHC staining score. The KM survival analysis revealed that the overall survival for patients in the PDLIM4^high^ group was significantly shorter than for those in the PDLIM4^low^ group (Fig. 1k). Clinical pathological characteristic is shown in Table 1. Subsequently, we conducted univariate Cox analysis and found that Lymph node metastasis, Tumor sizes, Depth of invasion, high PDLIM4 expression and TNM stage were prognostic factors for GC. What’s more, multivariate regression study demonstrated that PDLIM4 and tumor size served as independent prognostic indicators in GC (Table 2). Thus, we deduce that higher levels of PDLIM4 are associated with a negative prognosis and might function as an independent factor predicting outcomes in GC.

**Table 1:** Clinicopathological characteristic according to PDLIM4

Variables	N	PDLIM4^low ^(n = 105)	PDLIM4^high ^(n = 105)	*p*-value
Sex				0.144
Male	139	64 (46.0%)	75 (54.0%)	
Female	71	41 (57.7%)	30 (42.3%)	
Age (years)				0.333
< 60	98	53 (54.1%)	45 (45.9%)	
≥ 60	112	52 (46.4%)	60 (53.6%)	
Tumor size (cm)				< 0.001
< 4	112	69 (61.6%)	43 (38.4%)	
≥ 4	98	36 (36.7%)	62 (63.3%)	
Depth of invasion				0.002
T1 + T2	59	40 (67.8%)	19 (32.2%)	
T3 + T4	151	65 (43.0%)	86 (57.0%)	
Lymph node metastasis				0.539
N0	59	32 (54.2%)	27 (45.8%)	
N1 + N2 + N3	151	73 (48.3%)	78 (51.7%)	
TNM stage				0.023
I + II	81	49 (60.5%)	32 (39.5%)	
III + IV	129	56 (43.4%)	73 (56.6%)

**Table 2:** Univariate and multivariate COX regression analysis of prognostic factors associated with OS

Varibles	Univariate analysis		Multivariate analysis	
	HR (95% CI)	*p-*vable	HR (95% CI)	*p-*vable
Sex (female vs. male)	1.097 (0.763–1.578)	0.616		
Age (years) (≥ 60 vs. < 60)	1.117 (0.789–1.581)	0.534		
Tumor size (cm) (≥ 4 vs. < 4)	2.498 (1.751–3.563)	< 0.001	1.527 (1.042–2.236)	0.030
Depth of invasion (T2 + T3 vs. T1 + T2)	4.126 (2.470–6.892)	< 0.001	1.982 (0.870–4.514)	0.103
Lymph node metastasis (N1 + N2 + N3 vs. N0)	2.081 (1.343–3.225)	0.001	0.856 (0.316–2.317)	0.760
TNM stage (III + IV vs. I + II)	3.028 (2.014–4.551)	< 0.001	1.686 (0.539–5.278)	0.370
PDLIM4 (high/Low)	2.794 (1.934–4.037)	< 0.001	2.241 (1.543–3.254)	< 0.001

### PDLIM4 promotes malignant progression of GC cells in vitro and in vivo

**Fig. 2:**
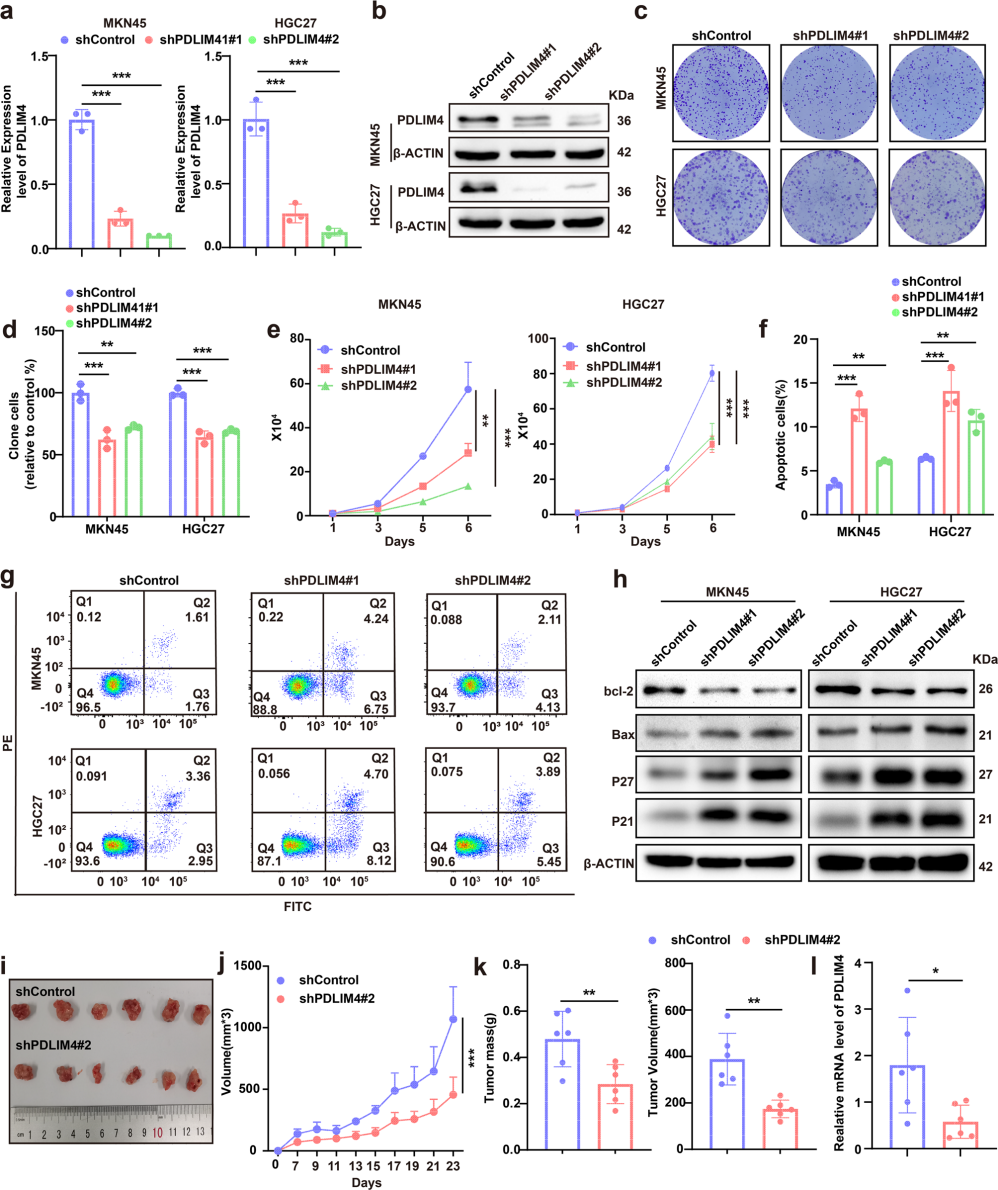
PDLIM4 promotes malignant progression of GC cells in vitro and in vivo.a RT-qPCR analysis demonstrated the efficiency of PDLIM4 KD at the mRNA level in MKN45 and HGC27 cells. b Western blotting analysis corroborated the knockdown efficiency of PDLIM4 at the protein level in MKN45 and HGC27 cells. c-d Clonal formation assays were conducted in MKN45 and HGC27 cells with PDLIM4 KD. e Cell viability assays indicated the effects of PDLIM4 depletion on MKN45 and HGC27 cells. f-g Apoptotic responses in MKN45 and HGC27 cells following PDLIM4 depletion were assessed. h The expression levels of apoptosis-related protein and cell cycle proteins were evaluated in MKN45 and HGC27 cells with PDLIM4 KD. i Representative images of tumors harvested from mice injected with shPDLIM4 or shControl MKN45 cells are presented. j Tumor growth was monitored in vivo, measuring tumor volume over time. k The weight and volume of harvested tumors were recorded (n = 6). l The mRNA expression levels of PDLIM4 in each tumor group were quantified. Data were presented as means±SD. *P<0.05; **P<0.01; ***P<0.001.

Given the pathological involvement of PDLIM4 in GC, we sought to explore its biological role in GC cells. We successfully established PDLIM4 knockdown (KD) GC cell lines in MKN45 and HGC27 (Fig. 2a, b). Next, we examined the influence of PDLIM4 on GC cell growth by conducting colony formation and cell viability assays. The results indicated that PDLIM4 KD markedly impeded the growth of MKN45 and HGC27 cells (Fig. 2c–e). Interesting, our findings indicated that the reduced viability of GC cells resulting from PDLIM4 KD was not solely due to impaired proliferative capacity, but also to the induction of apoptosis. Apoptosis experiment demonstrated that PDLIM4 KD promotes apoptosis in GC cells (Fig. 2f, g**)**. Consistently, changes in the cyclin related protein P21, P27 and apoptosis related protein Bax, bcl2 suggested that PDLIM4 KD inhibited cell proliferation and promoted apoptosis (Fig. 2h). We conducted a transwell assay to investigate whether PDLIM4 affects the metastatic potential of GC cells. PDLIM4 KD was found to impair the migratory ability of MKN45 and HGC27 cells (Figure S2a, c). Further evidence of metastasis related indicators also proves this conclusion (Figure S2b). We created a xenograft model to investigate the function of PDLIM4 in vivo. The results shown that PDLIM4 KD significantly reduced the tumors growth of BALB/c nude mice (Fig. 2i-k**)**. The knockdown efficiency of PDLIM4 in each group of tumors has been confirmed (Fig. 2l). Conversely, when PDLIM4 was overexpressed in MKN45 and HGC27 cells, the results were opposite both in vitro and in vivo (Figure S2d-l). All in all, our findings shown that PDLIM4 KD has an anti-tumor effect on GC cells.

### PDLIM4 interacts with HSP70

**Fig. 3:**
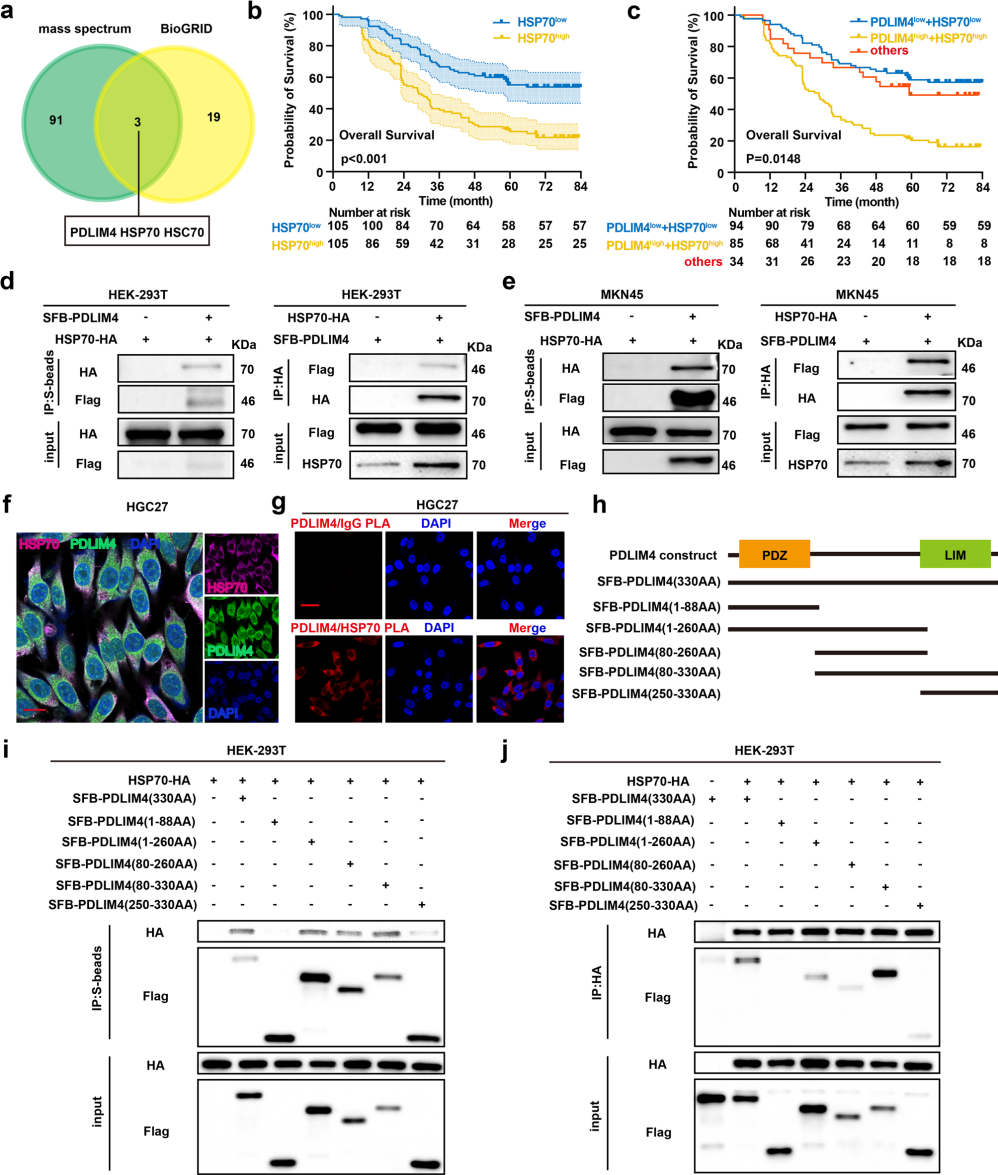
PDLIM4 interacts with HSP70.a Integration of mass spectrometry data with the BioGRID database. b Kaplan-Meier survival analysis of OS was performed using HSP70 expression levels. Patients were split into two groups, HSP70^High^ and HSP70^low^, based on the median IHC staining score (6 for HSP70). c Kaplan-Meier survival curves for OS in patients categorized by the IHC staining scores (5.33 for PDLIM4 and 6 for HSP70) are shown. Patients were grouped into three categories based on the expression levels of PDLIM4 and HSP70: PDLIM4^High^ + HSP70^High^, PDLIM4^Low^ + HSP70^Low^ and others. d Examination of the interaction between exogenously expressed SFB-tagged PDLIM4 and HSP70 in HEK-293T cells using a co-immunoprecipitation assay. e Investigation of the interaction between exogenously expressed SFB-tagged PDLIM4 and HSP70 in MKN45 cells via a co-immunoprecipitation assay. f Immunofluorescence analysis of PDLIM4 and HSP70 expression in HGC27 cells, Scale bar: 20 μm. g The interaction between PDLIM4 and HSP70 in HGC27 cells was detected by PLA, Scale bar: 20μm. h Based on previous studies, PDLIM4 is categorized into distinct structural domains. i-j Analysis of the interaction between various exogenous SFB-tagged PDLIM4 constructs (330AA, 1-88AA, 1-260AA, 80-260AA, 80-330AA, 250-330AA) and HSP70 in HEK-293T cells through a co-immunoprecipitation (CO-IP) assay.

To understand the mechanism responsible for PDLIM4-mediated malignant progression of GC, we performed mass spectrometry analysis to identify proteins interacting with PDLIM4. The proteins identified through mass spectrometry were cross-referenced with predicted proteins in the BioGRID database, revealing HSP70 and HSC70 as potential binding partners (Fig. 3a). The GSE122401 datebase indicated a correlation between PDLIM4 and HSP70, rather than HSC70 (Figure S3a-b). Moreover, we discovered that GC tissues exhibited elevated mRNA and protein levels of HSP70 when compared to the adjacent normal tissues (Figure S3c-d). Notably, GC patients exhibiting high HSP70 expression had a poorer OS (Fig. 3b). Survival analysis further indicated that patients with elevated expression of both PDLIM4 and HSP70 had shorter overall survival, whereas the most favorable prognosis was observed in patients with low expression of both PDLIM4 and HSP70 (Fig. 3c). Based on these findings, HSP70 was selected for further detailed investigation. Co-immunoprecipitation (Co-IP) assays were conducted in HEK-293 T cells to confirm the interaction between PDLIM4 and HSP70. HEK-293 T cells transfected with exogenous SFB-PDLIM4 or HSP70-HA plasmids, the results demonstrated that PDLIM4 interacts with HSP70 exogenously **(**Fig. 3d**)**. This interaction was further corroborated in MKN45 cells using the same experimental (Fig. 3e). Immunofluorescence analysis revealed the co-localization of PDLIM4 and HSP70 within the cytoplasm (Fig. 3f, Figure S3e). The PLA experiments also revealed the co-localization of PDLIM4 and HSP70 in HGC27 and MKN45 cells (Fig. 3g, Figure S3f). In addition, we also depicted the binding domains of PDLIM4 and HSP70 in HEK-293 T cells transfected with PDLIM4 truncated mutants and HSP70-HA. The research results indicate that only the N-terminal fragment containing the PDZ domain failed to interact with HSP70, while all other fragments bound to HSP70 (Fig. 3h–j). Collectively, these results imply that PDLIM4 binds to HSP70 through its C-terminal and intermediate regions.

### PDLIM4 positive regulates HSP70’s protein level through ubiquitination

**Fig. 4:**
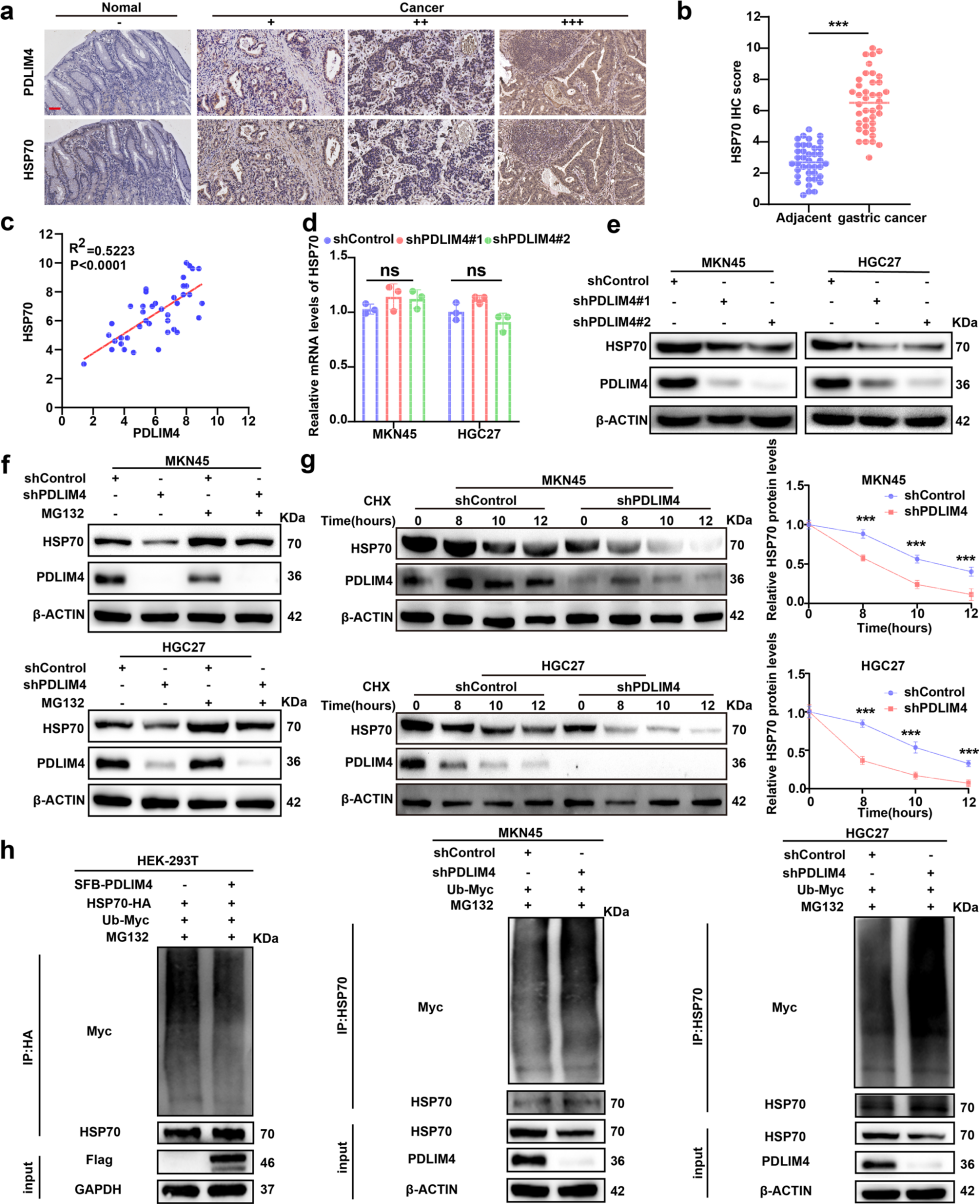
PDLIM4 positive regulates HSP70’s Protein level through ubiquitination. a Representative immunohistochemistry staining images of PDLIM4 and HSP70 across various grades of GC tissues and adjacent normal tissues, scale bars: 50μm. b IHC scores for HSP70 in 40 pairs of samples of GC tissues and adjacent normal tissues. c Analysis of the protein expression levels of PDLIM4 and HSP70 in GC tissues using Spearman 's correlation. d Evaluation of the mRNA expression level of HSP70 in GC cells following PDLIM4 KD. e Assessment of the protein expression level of HSP70 in GC cells with PDLIM4 KD. f Western blotting analysis of HSP70 in MKN45 and HGC27 cells with PDLIM4 KD after treatment with MG132 (20 μM). g Determination of the HSP70 degradation half-life in MKN45 and HGC27 cells with PDLIM4 KD in the presence of cycloheximide (CHX, 200 μg/mL). h Analysis of HSP70 ubiquitination levels in cells with PDLIM4 knockdown or overexpression. Data were presented as means±SD. ***P<0.001; ns, no statistical difference.

To investigate the specific mechanism of PDLIM4 mediated regulation of HSP70, we performed immunohistochemistry on fresh GC tissues, and found that PDLIM4 is positive correlated with HSP70 at the Protein level (Fig. 4a–c). Subsequently, we utilized western blotting and RT-qPCR to measure the protein and mRNA levels of HSP70 in MKN45 and HGC27 cells with PDLIM4 KD, the finding indicated that PDLIM4 influences HSP70 Protein expression at the post-transcriptional level, while not transcription level (Fig. 4d, e). Accordingly, we theorized that PDLIM4 regulates the HSP70 Protein level through its degradation pathway. After that treatment with a proteasome inhibitor MG132, which caused a notably increase at the protein levels of HSP70 in PDLIM4-KD MKN45 and HGC27 cells (Fig. 4f). This data suggests that HSP70 is consistently degraded by a ubiquitin–proteasome-dependent pathway in GC. We carried out a cycloheximide (CHX) chase assay to further investigate the impact of PDLIM4 on HSP70 stability by observing its degradation kinetics. According to our results, there was a significant decline in the half-life of HSP70 in PDLIM4 KD (Fig. 4g). The two experiments mentioned above indicated that PDLIM4 might hinder the degradation of HSP70 through the proteasomal pathway. This hypothesis was supported by our finding that inducing PDLIM4 expression reduced HSP70 ubiquitination in HEK-293 T cells and increased ubiquitination level of HSP70 in PDLIM4 KD MKN45, HGC27 cells (Fig. 4h). In conclusion, our research provided a proof that PDLIM4 modulates the HSP70 Protein stability in GC cells by regulating its ubiquitination and proteasomal degradation.

### PDLIM4 blocks STUB1 interaction with HSP70 and STUB1-mediated ubiquitination and proteasomal degradation of HSP70

**Fig. 5:**
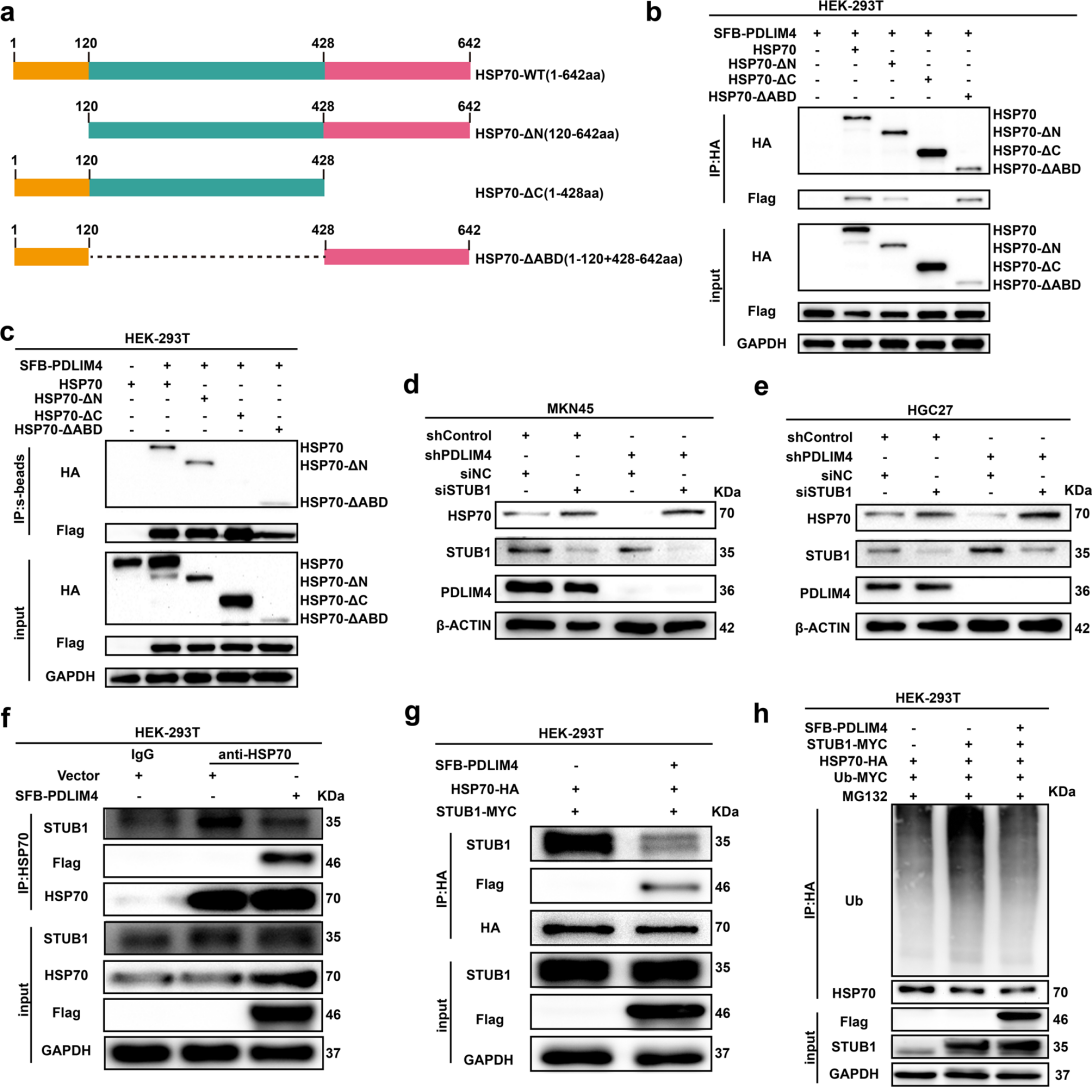
PDLIM4 blocks STUB1 interaction with HSP70 and STUB1-mediated ubiquitination and proteasomal degradation of HSP70.a Previous studies have delineated that HSP70 comprises several distinct structural domains. b-c The interaction among exogenous HSP70, HSP70-ΔN, HSP70-ΔC, HSP70-ΔABD and SFB-PDLIM4 in HEK-293T cells was examined using a co-immunoprecipitation (CO-IP) assay. d-e. Western blotting analyses were conducted to assess the expression levels of PDLIM4, HSP70, and STUB1 in MKN45 and HGC27 cells, which were co-transfected with PDLIM4 shRNA and STUB1 siRNA. f The presence of exogenous SFB-PDLIM4 disrupted the interaction between endogenous HSP70 and STUB1 in HEK-293T cells. g Exogenous SFB-PDLIM4 disrupted the interaction between exogenous HSP70 and STUB1 in HEK-293T cells. h The levels of HSP70 ubiquitination were evaluated in HEK-293T cells with overexpressing STUB1 or co-expressing both STUB1 and PDLIM4.

Given that neither PDLIM4 nor HSP70 are genes associated with ubiquitination, we speculate whether there is an E3 ubiquitin ligase or deubiquitinase involved in function. Consequently, we carried out an HSP70 deletion mapping experiments in an exogenous system to identify the HSP70 region that associates with PDLIM4 (Fig. 5a). SFB-PDLIM4 was transfected into HEK-293 T cells together with HSP70 deletion mutants. Co-immunoprecipitation (Co-IP) experiments using protein lysates from these transfected cells demonstrated that PDLIM4 interacts with the C-terminal region of HSP70 (Fig. 5b, c). Earlier studies have demonstrated that STUB1 serves as an E3 ubiquitin ligase that ubiquitinates HSP70 by binding to its C-terminus. Our experiments also confirmed this conclusion (Figure S5a-c). Our findings suggest that both PDLIM4 and STUB1 associate with the C-terminal region of HSP70, indicating a potential competitive binding model. To further investigate this hypothesis, we examined whether STUB1 knockdown (KD) could alter the impact of PDLIM4 KD on HSP70 protein levels. The results showed that STUB1 KD could reverse the effect of PDLIM4 KD on HSP70 protein reduction in MKN45 and HGC27 cells (Fig. 5d, e), thereby highlighting that the regulation of HSP70 by PDLIM4 is mediated through STUB1. Subsequently, we demonstrated that overexpression of SFB-PDLIM4 can disrupt the endogenous interaction between STUB1 and HSP70 in HEK-293 T cells (Fig. 5f). Furthermore, we performed in vitro binding assays using lysates from HEK-293 T cells individually transfected with HSP70-HA, STUB1-MYC, and SFB-PDLIM4. Immunoprecipitation of HSP70 followed by western blotting analysis showed that the overexpression of SFB-PDLIM4 reduced the interaction between STUB1 and HSP70 (Fig. 5g). These experiments collectively support a competitive binding model. To evaluate the impact of PDLIM4 on STUB1-mediated ubiquitination of HSP70, we conducted in vitro ubiquitination assays using translated HSP70-HA, STUB1-MYC, and SFB-PDLIM4. We confirmed that STUB1 ubiquitinates HSP70 in vitro, whereas the addition of SFB-PDLIM4 effectively inhibited STUB1-mediated polyubiquitination of HSP70 (Fig. 5h). Consistent with these biochemical results, our findings suggest that the inhibition of GC progression due to PDLIM4 KD can be reversed by STUB1 knockdown (Figure S5d-j). These findings collectively suggest that STUB1 is a genuine E3 ubiquitin ligase targeting HSP70 in GC, and PDLIM4 directly competed with STUB1 to bind with HSP70, leading to down-regulated ubiquitination of HSP70.

### PDLIM4 promotes GC cells malignant progression by regulating the HSP70-mediated MAPK signaling pathway

**Fig. 6:**
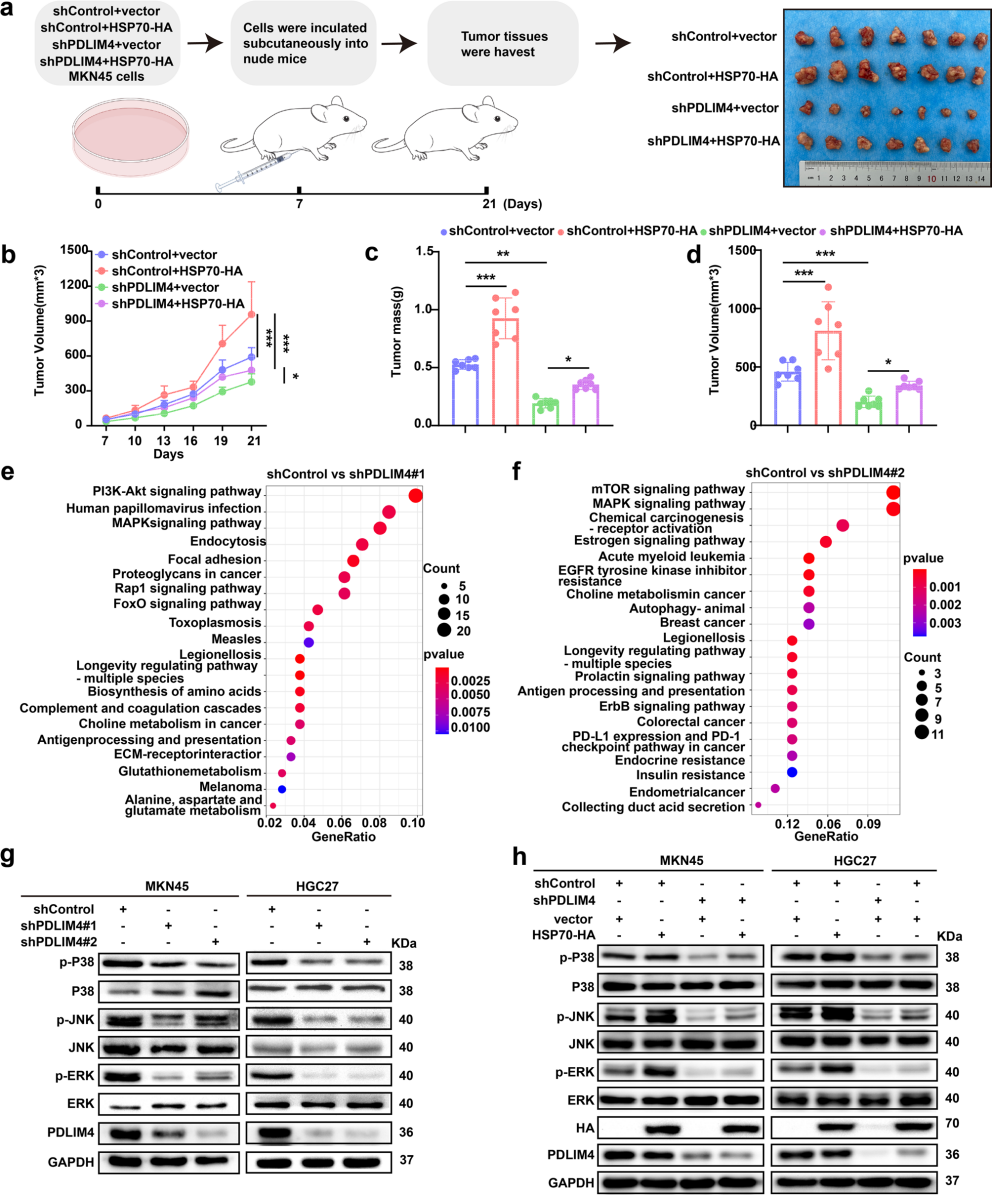
DLIM4 promotes GC cells malignant progression by regulating the HSP70-mediated MAPK signaling pathway.a Diagrammatic representation of the in vivo and image of tumors. b Growth of tumor volume in vivo. c-d Weight and volume of the harvested tumors (n = 7). e-f KEGG enrichment analysis of differentially expressed genes between shControl and shPDLIM4#1/2 MKN45 cells. g Western blotting was used to analyze the total and phosphorylation protein levels of P38, JNK, and ERK on MKN45 and HGC27 cells with PDLIM4 KD. h Western blotting was used to analyze the total and phosphorylation protein levels of P38, JNK, and ERK on PDLIM4 KD MKN45 and HGC27 cells with HSP70 overexpression. Data were presented as means±SD. *P<0.05; **P<0.01; ***P<0.001.

Since HSP70 has been established as a poor prognostic factor in GC, its role in PDLIM4 KD GC cells remains unclear. To elucidate the function of HSP70 in PDLIM4 KD GC cells, we successfully overexpressed HSP70 in PDLIM4 KD MKN45 and HGC27 cell lines (Figure S6a-b). In vitro experiments demonstrated that HSP70 overexpression counteracts the inhibitory effects of PDLIM4 KD on the proliferation and metastasis of GC cells (Figure S6c-g) and mitigates the pro-apoptotic effects induced by PDLIM4 KD (Figure S6h-i). Additionally, we investigated whether PDLIM4-mediated cell growth is dependent on HSP70 in vivo (Fig. 6a). Subcutaneous xenograft experiments indicated that the reduction in tumorigenesis caused by PDLIM4 KD in MKN45 cells was significantly reversed by HSP70 overexpression (Fig. 6b–d). The results indicated that the suppression of malignant progression in GC by PDLIM4 KD is contingent upon HSP70. Furthermore, KEGG enrichment analysis of PDLIM4 KD MKN45 cells revealed a significant enrichment in the MAPK pathway (Fig. 6e, f). Western blotting analysis showed that reducing the protein expression of PDLIM4 in GC cells led to the suppression of the MAPK signaling pathway, as demonstrated by diminished phosphorylation levels of P38, JNK, and ERK (Fig. 6g). Moreover, overexpression of HSP70 in PDLIM4 KD MKN45 and HGC27 cells resulted in a partial reactivation of the MAPK signaling pathway (Fig. 6h). Overall, PDLIM4 contributes to the GC cells' malignant progression by regulating the MAPK signaling pathway via HSP70.

### PDLIM4 enhances GC cell resistance to DDP in a manner of HSP70

**Fig. 7:**
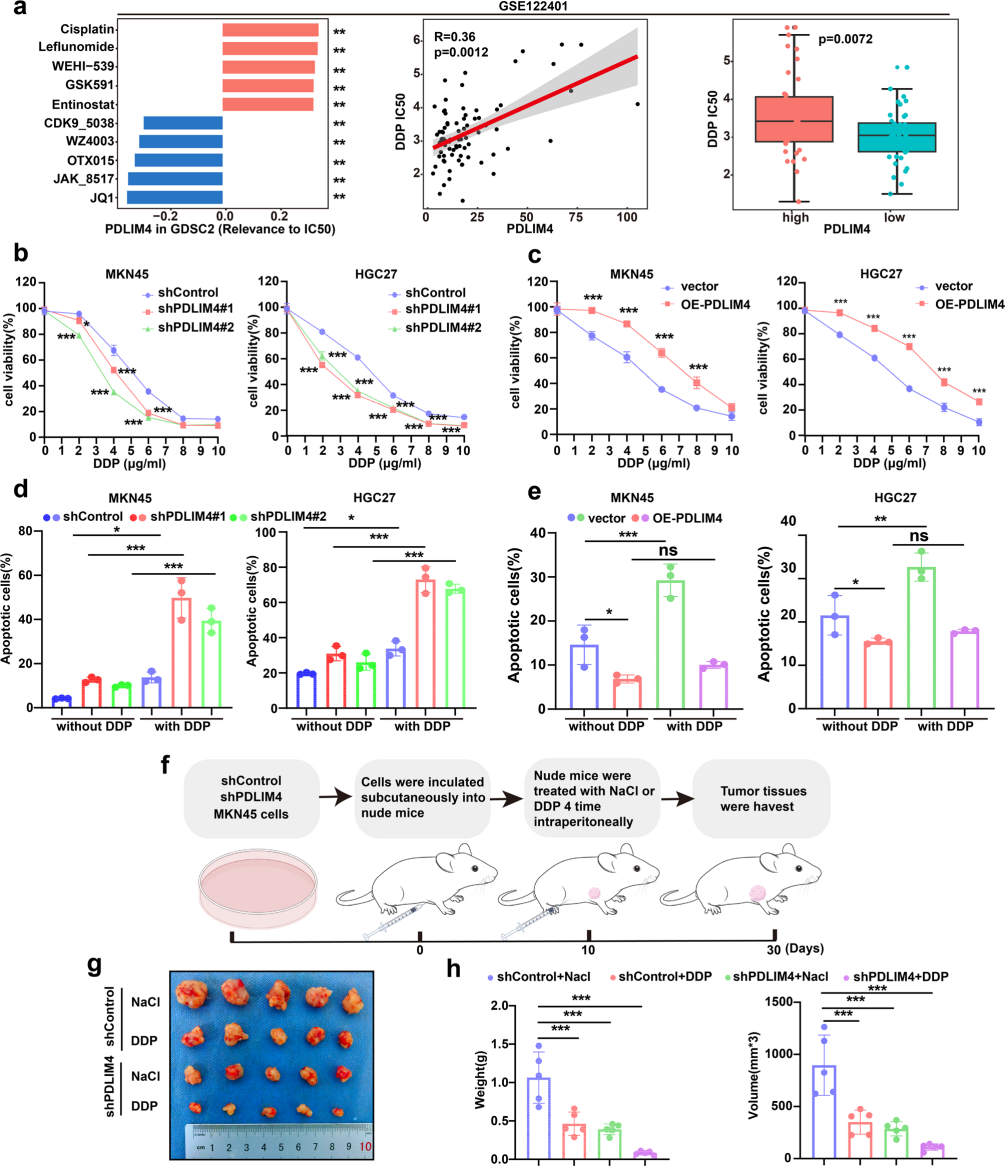
PDLIM4 enhances GC cell resistance to DDP in a manner of HSP70.a Analysis of the correlation between PDLIM4 expression levels and DDP IC50 values in the GSE122401 dataset. b Assessment of cell viability in PDLIM4 KD cells following a 24-hour treatment with DDP. c Assessment of cell viability in PDLIM4 overexpression cells following a 24-hour treatment with DDP. d Examination of apoptotic activity in PDLIM4 KD MKN45 and HGC27 cells after 24 hours of DDP exposure. e Examination of apoptotic activity in PDLIM4 overexpression MKN45 and HGC27 cells after 24 hours of DDP exposure. f Diagrammatic representation of the in vivo. g Image of tumors. h Weight and volume of the harvested tumors (n = 5). Data were presented as means±SD. *P<0.05; **P<0.01; ***P<0.001; ns, no statistical difference.

Due to the widespread challenge of drug resistance in clinical chemotherapy, our research investigated the possible role of PDLIM4 in modulating chemotherapy drug resistance. We conducted a drug sensitivity analysis utilizing data from the GDSC and GSE122401 datasets. The Spearman correlation analysis showed a positive relationship between PDLIM4 expression levels and the IC50 values of DDP. Furthermore, the IC50 values for DDP were significantly increased in the PDLIM4^high^ group relative to the PDLIM4^low^ group (Fig. 7a). Then we sought to determine whether PDLIM4 enhances GC cell resistance to DDP. Cell viability assays demonstrated that PDLIM4 KD indeed sensitized GC cells to DDP, but PDLIM4 Overexpression promoted GC cells resistance to DDP (Fig. 7b, c). An annexin-V/propidium iodide (PI) assay also proved this conclusion (Fig. 7d, e). To examine the role of PDLIM4 in modulating sensitivity to DDP in vivo, we administered MKN45 cells expressing either shControl or shPDLIM4 into the axillary region of nude mice. Once the average size of the xenografts in each group reached approximately 100 mm^3^, the mice were subjected to DDP treatment (at a dosage of 4 mg/kg, every five days for a total of four cycles) (Fig. 7f). The results indicated that PDLIM4 KD and DDP treatment inhibits tumor growth. Importantly, the combination of PDLIM4 KD and DDP treatment produced a pronounced synergistic effect, leading to a substantial decrease in tumor size (Figs. 7g, h). To further elucidate whether the resistance of PDLIM4-KD GC cells dependent on HSP70. DDP was exposed in PDLIM4-KD MKN45 and HGC27 cells with overexpressing of HSP70. Cell viability experiments, Cell cloning experiments, and apoptosis experiments all demonstrated that the overexpression of HSP70 can partially mitigate the sensitivity of PDLIM4 KD GC cells to DDP (Figures S7a-e). Our research proved that the combination of PDLIM4 KD and DDP yields a synergistic effect relies on HSP70. Additionally, we conducted some phenotype experiments on DDP-resistant GC cell lines AGS. We purchased a DDP resistant gastric cancer cell line AGS from Jiangsu Keygen Biotech Corp., Ltd.‌. Firstly, we conducted resistance testing on AGS sensitive and AGS resistant cells. 3 × 10^3^AGS or AGS/DDP cells were seeded into 96 well plate and treated with different concentrations (0, 0.15625, 0.3125, 0.625, 1.25,2.5 5,10, 20ug/ml) of DDP for 72 h. After 2 h of reaction with CCK8 reagent, the absorbance values were measured. The results showed that the construction of AGS resistant cells was successful (Figure S7f). Next, we detected the mRNA expression of PDLIM4 in AGS resistant and AGS sensitive cells, the final results indicated that the expression level of PDLIM4 was significantly higher in resistant cells than in sensitive cells (Figure S7g), which is consistent with our previous conclusion that the higher expression of PDLIM4 in GC, the lower sensitivity to DDP. Subsequently, we knocking down of PDLIM4 in AGS resistant cells (Figure S7h-i) and apoptosis (Figure S7j-k), CCK8 (Figure S7l) experiments have all confirmed that PDLIM4 KD in AGS resistant cells can reverse resistance to DDP. Overall, these experiments results indicated that Targeting PDLIM4 could represent a hopeful plan to enhance the sensitivity of GC treatment to DDP.

### The biological feature of siPDLIM4/DDP LNPs

**Fig. 8:**
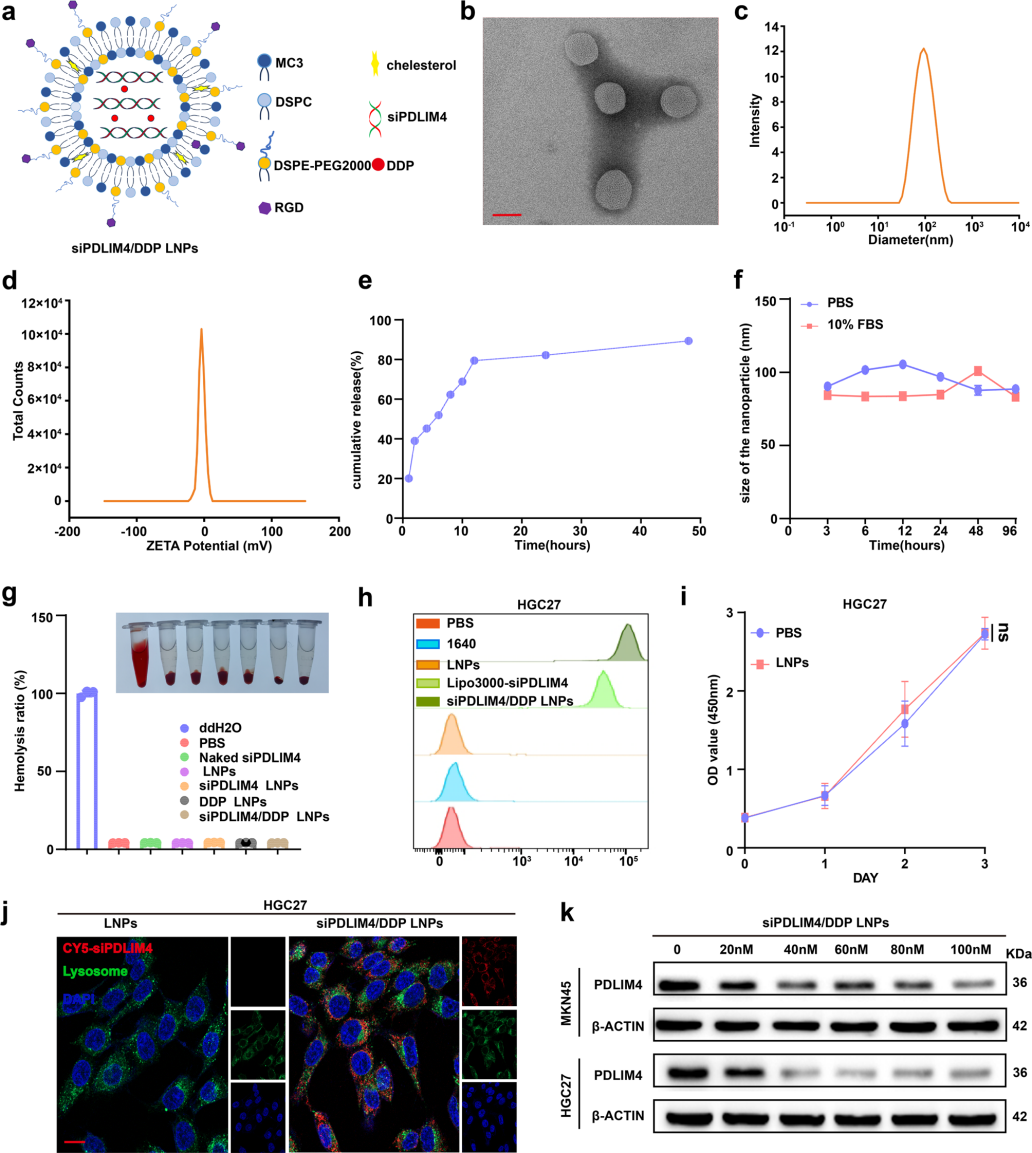
The Biological Feature of siPDLIM4/DDP LNPs.a A schematic representation of siPDLIM4/DDP LNPs is provided. b Transmission electron microscopy images of siPDLIM4/DDP LNPs are shown, scale bars: 100 nm. c-d Dynamic light scattering was employed to determine the particle sizes (c) and zeta potentials (d) of the siPDLIM4/DDP LNPs. e Cumulative release of DDP in siPDLIM4/DDP LNPs at different time. f The stability of siPDLIM4/DDP LNPs was assessed over a period of 96 hours in PBS or PBS containing 10% serum at 37 ℃. g Hemolysis of erythrocytes was observed following a 2-hour incubation with PBS, deionized water, or various LNPs. h The proportion of CY5-positive cells in HGC27 cells was quantified using flow cytometry. i The survival rate of HGC27 cells was evaluated after exposure to PBS or LNPs. j Confocal laser scanning microscopy images of HGC27 cells were acquired following 4-hour incubation with either LNPs or siPDLIM4/DDP LNPs. Endosomes were stained green with Lysotracker, nuclei were stained blue with Hoechst 33342, and siPDLIM4 was labeled with CY5, scale bars: 20μm. k The knockdown efficiency of PDLIM4 in MKN45 and HGC27 cells was detected by Western blotting after treatment with various concentrations of siPDLIM4/DDP LNPs. Data were presented as means±SD. ns, no statistical difference.

To verify the anti-tumor effect of siPDLIM4 in vivo and its potential to enhance the anti-tumor efficacy of DDP, we synthesized various lipid nanoparticles (LNPs), including LNPs, siPDLIM4 LNPs, DDP LNPs and siPDLIM4/DDP LNPs (Fig. 8a). Recently, many literatures [38–40] confirmed that gastric cells express α v β 3 and can specifically bind to RGD. To further confirm this conclusion, we validated the protein expression level of α v β 3 on MKN45 and HGC27 cells. Western blotting results showed that α v β 3 was expressed on MKN54 and HGC27 cells (Figure S8a), therefore, we added RGD to the nanoparticles to enhance its targeting ability towards gastric cancer cells. Initially, we characterized the physicochemical properties of the LNPs loaded with siPDLIM4 and DDP. Transmission electron microscopy (TEM) analysis revealed that the siPDLIM4/DDP LNPs were spherical (Fig. 8b), with an average diameter of approximately 86.01 nm **(**Fig. 8c**)** and a zeta potential of −5.45 mV (Fig. 8d). The encapsulation efficiency of siPDLIM4 was found to be approximately 98.18% by labeling it with the fluorescent dye CY5, referred to as CY5-siPDLIM4. We conducted a drug release curve in PH6.0 PBS, which is a tumor-mimicking environments (Fig. 8e). In the presented result we can see that approximate 80% of DDP was released at 12 h, the cumulative release of DDP reached more than 90% at 48 h. The results indicated that the drug release of siPDIM4/DDP LNPs was successful. Furthermore, the stability of siPDLIM4/DDP LNPs was assessed at different time intervals up to 96 h, and the findings indicated that the nanoparticles size remained constant over time **(**Fig. 8f**)**. The stability, safety, and high transfection efficiency of LNPs have been substantiated through our experimental investigations. An essential criterion for assessing the biocompatibility of materials is their blood compatibility. Visual inspection indicated significant hemolysis in the positive control group, whereas both the LNPs group and the negative control groups exhibited no discernible hemolysis. Quantitative analysis conducted using a microplate reader consistently demonstrated that the hemolysis rates for all LNPs groups remained below 5% (Fig. 8g). Compared to the Lip3000-transfected group, the siPDLIM4/DDP LNPs group exhibited a significantly greater proportion of CY5-positive cells (Fig. 8h, Figure S8b). The in vitro cytotoxicity of nanoparticles was assessed by applying LNPs and PBS to MKN45 and HGC27 cells over a 72-h period, revealing no remarkably differences in cell viability between the LNPs and PBS groups (Fig. 8i, Figure S8c). Successful gene delivery and expression were contingent upon high uptake levels and efficient lysosomal release. Upon incubating CY5-siPDLIM4/DDP LNPs and LNPs with MKN45 and HGC27 cells for a duration of four hours, for CY5-siPDLIM4/DDP LNPs, the majority of the siPDLIM4 (indicated by red fluorescence) successfully escaped from the endosomes (indicated by green fluorescence) and translocated into the cytoplasm, where it exerted its function. In contrast, the LNPs exhibited minimal fluorescence (Fig. 8jand Figure S8d). Owing to their effective endosomal escape capability, the siPDLIM4/DDP LNPs markedly reduced PDLIM4 expression in MKN45 and HGC27 cells, achieving near complete suppression at a siRNA concentration of 40 nM (Fig. 8k and Figure S8e). The experimental results collectively demonstrate that the siPDLIM4/DDP LNPs were successfully constructed, exhibiting high transfection efficiency, substantial cellular uptake, and a favorable safety profile.

### siPDLIM4/DDP LNPs inhibit the malignant progression of GC cells and increase the sensitivity to DDP treatment in vitro

**Fig. 9:**
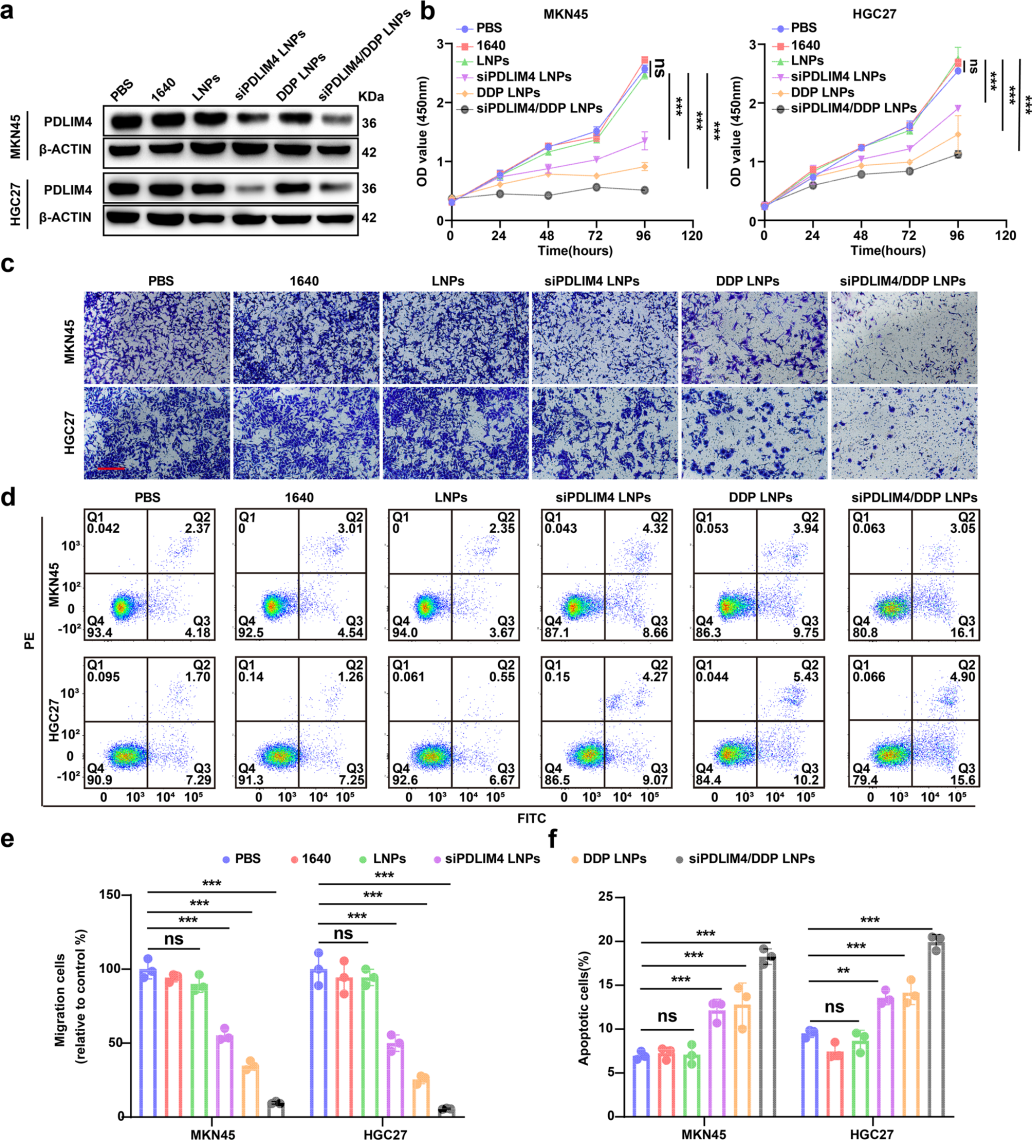
siPDLIM4/DDP LNPs inhibit the malignant progression of GC cells and increase the sensitivity to DDP treatment in vitro.a The protein expression levels of PDLIM4 were assessed in MKN45 and HGC27 cells using western blotting following treatment with PBS, 1640, LNPs, siPDLIM4 LNPs, DDP LNPs, or siPDLIM4/DDP LNPs. b The cell viability of MKN45 and HGC27 cells was evaluated after treatment with PBS, 1640, LNPs, siPDLIM4 LNPs, DDP LNPs, or siPDLIM4/DDP LNPs. c, e The migratory capacity of MKN45 and HGC27 cells, treated with PBS, 1640, LNPs, siPDLIM4 LNPs, DDP LNPs, or siPDLIM4/DDP LNPs, was determined using an in vitro Transwell migration assay, scale bars: 200μm. d, f Apoptosis in MKN45 and HGC27 cells was analyzed following treatment with PBS, 1640, LNPs, siPDLIM4 LNPs, DDP LNPs, or siPDLIM4/DDP LNPs. Data were presented as means±SD. **P<0.01; ***P<0.001; ns, no statistical difference.

Upon confirming the biological characteristics of siPDLIM4/DDP LNPs, we investigated their therapeutic effects on GC cells in vitro. As illustrated in Fig. 8j, siPDLIM4/DDP LNPs were showed to inhibit over 70% of PDLIM4 expression at an siRNA concentration of 40 nM. Consequently, this siRNA dosage was selected for subsequent experiments. Western blotting analysis further revealed that both the siPDLIM4/DDP LNPs and siPDLIM4 LNPs groups significantly reduced PDLIM4 expression compared to the PBS, 1640, LNPs, and DDP LNPs groups (Fig. 9a). Cell proliferation and migration experiments demonstrated that both the siPDLIM4 LNPs and DDP LNPs groups significantly inhibited GC cell growth and migration compared with the control group, with the siPDLIM4/DDP LNPs group exhibiting an enhanced inhibitory effect (Fig. 9b, c, e). Additionally, an Annexin-V/propidium iodide (PI) assay demonstrated that the combination of siPDLIM4 and DDP LNPs significantly enhanced apoptotic activity (Fig. 9d, f). Overall, siPDLIM4 LNPs and DDP LNPs demonstrate a significant capacity to suppress the proliferation and metastasis of GC cells, while also promoting apoptosis. Moreover, the combined application of siPDLIM4/DDP LNPs further amplifies these effects and enhances the sensitivity of GC cells to DDP in vitro.

### In vivo evaluation of the therapeutic efficacy and toxicity of siPDLIM4/DDP LNPs

**Fig. 10:**
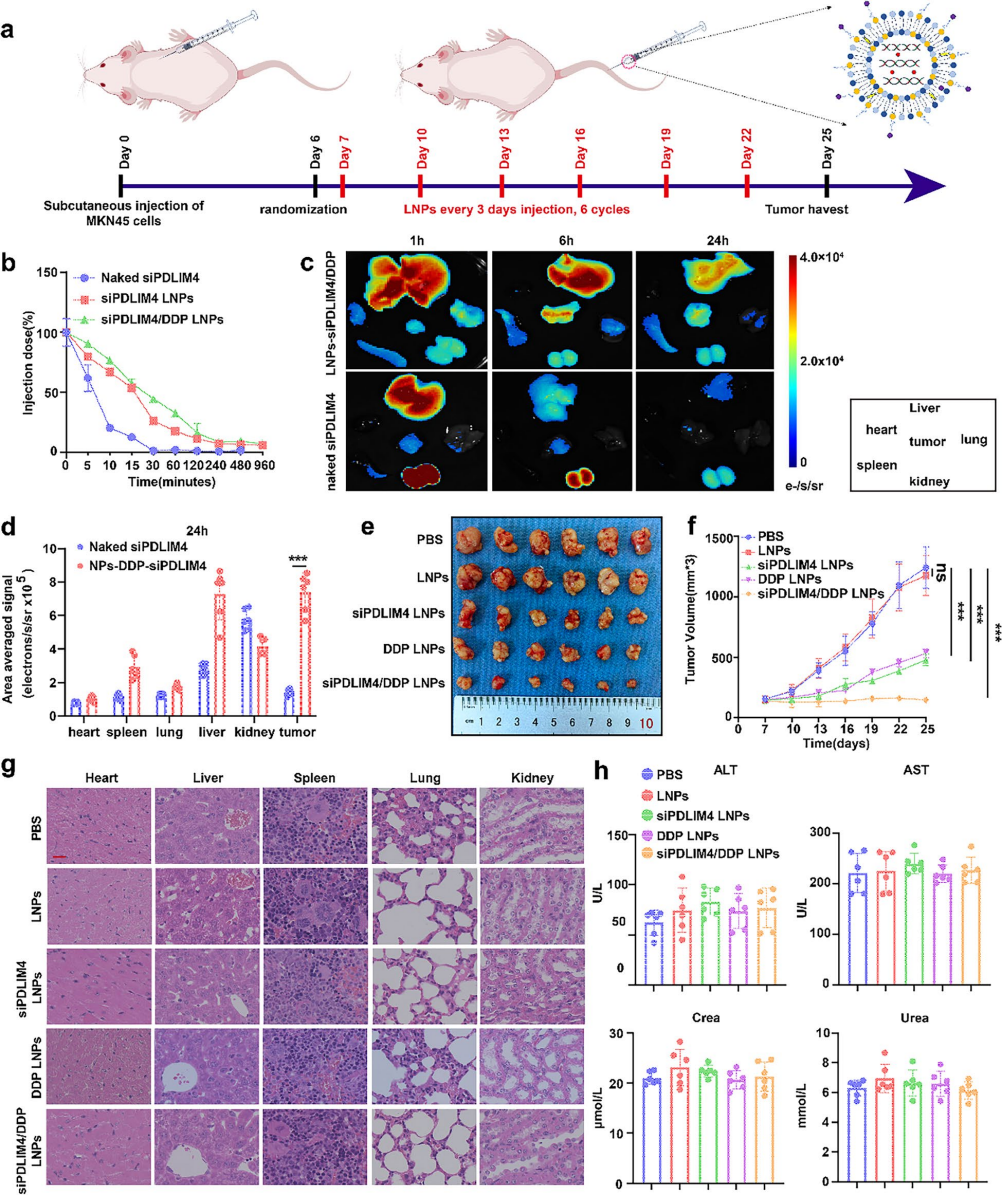
In vivo evaluation of the therapeutic efficacy and toxicity of siPDLIM4/DDP LNPs.a A schematic diagram illustrates the timeline for tumor implantation and the administration of PBS, LNPs, siPDLIM4 LNPs, DDP LNPs, and siPDLIM4/DDP LNPs in mice bearing MKN45 tumors. The mice received LNPs injections at three-day intervals for a total of six cycles. b The pharmacokinetics of naked siPDLIM4, siPDLIM4 LNPs and siPDLIM4/DDP LNPs were evaluated in vivo. c Naked CY5-siPDLIM4 and CY5-siPDLIM4/DDP LNPs were administered intravenously and 1, 6, 24 hours post-injection, tumors and major organs were harvested for biodistribution analysis using small animal CT/live imaging all-in-one machine (Milabs B.V.). d Quantitative data revealed the distribution of naked siPDLIM4 and siPDLIM4/DDP LNPs across various organs, including tumors, in MKN45 tumor bearing mice after 24 hours post-injection. e Representative images of tumors (n = 6) are provided. f The progression of tumor volume in vivo is depicted. g Hematoxylin and eosin (H&E) stained images of key organs are presented following treatment with PBS, LNPs, siPDLIM4 LNPs, DDP LNPs, and siPDLIM4/DDP LNPs, scale bar: 20 μm. h Serum levels of ALT, AST, creatinine, and urea were measured following treatment with PBS, LNPs, siPDLIM4 LNPs, DDP LNPs, and siPDLIM4/DDP LNPs. Data were presented as means±SD. ***P<0.001; ns, no statistical difference.

Subsequently, we conducted a comprehensive investigation into the pharmacokinetics (PK) and biodistribution (BioD) of siPDLIM4/DDP LNPs. To this end, healthy mice were administered intravenous injections of naked CY5-siPDLIM4, CY5-siPDLIM4/DDP LNPs, and CY5-siPDLIM4 LNPs, at a dosage of 1 nmol siRNA (n = 3). As illustrated in Fig. 10b, the naked siPDLIM4 was rapidly cleared from the bloodstream, whereas both siPDLIM4/DDP LNPs and siPDLIM4 LNPs demonstrated prolonged half-lives, with approximately 10% of the initial dose remaining detectable two hours post-injection. Subsequently, to evaluate the biodistribution, a xenograft model harboring MKN45 cell derived tumors in the axillary region was utilized. Naked CY5-siPDLIM4 and CY5-siPDLIM4/DDP LNPs were administered intravenously, and 1, 6, 24 h post-injection, tumors and major organs were harvested for biodistribution analysis. Naked siRNA is mainly concentrated in the kidneys, followed by the liver, and almost no fluorescence is observed in tumor tissues. However, CY5-siPDLIM4/DDP LNPs show significantly higher tumor accumulation than naked siRNA. The fluorescence intensity of the CY5-siPDLIM4/DDP LNPs in the kidneys and liver subsequently decreased, indicating that the lipid-based delivery system protects CY5-siPDLIM4/DDP from clearance by the kidneys and liver, thereby improving the circulation time of CY5-siPDLIM4/DDP in the body. The results revealed that CY5-siPDLIM4/DDP LNPs exhibited significantly higher fluorescence intensity at the tumor compared to the naked CY5-siPDLIM4 at multiple time points (Fig. 10c, d and Figure S10a). In addition, after injecting naked siPDLIM4 and CY5-siPDLIM4/DDP LNPs for 24 h, we removed the tumor tissue for validation the expression of PDLIM4. We found that the CY5- siPDLIM4/DDP LNPs group could knock down the expression of PDLIM4 compared with the naked siPDLIM4 group (Figure S10b). This result further confirmed that CY5siPDLIM4/DDP LNPs can specifically target tumor tissue and exert the effect of knocking down PDLIM4, while the naked siPDLIM4 cannot target tumor tissue, which is consistent with the fluorescence results. To further assess the anti-tumor efficacy of siPDLIM4/DDP LNPs, a xenograft tumor model was established by injecting MKN45 cells into the axillary region of mice. Subsequently, PBS, LNPs, siPDLIM4 LNPs, DDP LNPs, and siPDLIM4 DDP LNPs were administered via tail vein injection into mice every three days, totaling six injections (Fig. 10a). In comparison to the PBS and LNPs groups, the groups treated with siPDLIM4 LNPs and DDP LNPs exhibited a reduction in tumor volume. Notably, the siPDLIM4/DDP LNPs group demonstrated the smallest tumor volume, indicating that both siPDLIM4 LNPs and DDP LNPs possess anti-tumor properties, and their combined use results in a synergistic effect **(**Fig. 10e, f and Figure S10c). The treatment process did not result in significant body weight differences among the mice in each group, suggesting the safety of the treatment (Figure S10d). Immunohistochemical analysis confirmed that the protein levels of PDLIM4 in the siPDLIM4 LNPs and siPDLIM4/DDP LNPs groups were significantly reduced compared to the other three groups, indicating successful in vivo knockdown of PDLIM4 expression. Furthermore, Ki67 assays corroborated that both siPDLIM4 LNPs and DDP LNPs exhibited anti-tumor effects, with siPDLIM4/DDP LNPs demonstrating synergistic anti-tumor advantages. Additionally, Immunohistochemical analysis confirmed that the protein levels of HSP70 in the siPDLIM4 LNPs and siPDLIM4/DDP LNPs groups were significantly reduced compared to the other three groups, indicating that after knocking down of PDLIM4, the protein level of HSP70 also decreased (Figure S10e-h). H&E staining was employed to assessed systemic toxicity, revealing no significant damage to the heart, liver, spleen, lungs, and kidneys of mice treated with different LNPs compared to the PBS group (Fig. 10g). Additionally, assays for AST, ALT, creatinine, and urea excluded the presence of hepatotoxicity and nephrotoxicity (Fig. 10h). In conclusion, siPDLIM4/DDP LNPs exhibited significant anti-tumor properties without inducing notable toxicity in major organs in vivo, providing substantial evidence for potential clinical application.

## Discussion

The malignant progression and chemoresistance of GC can result from the excessive activation of abnormal signaling pathways, which hinders effective treatment. In this study, we demonstrated that PDLIM4 inhibits the ubiquitination and proteasomal degradation of HSP70 by directly blocking the interaction between STUB1 and HSP70, thereby activating the MAPK signaling pathway. This activation ultimately facilitated the malignant progression and DDP resistance of GC cells. To translated this discovery into clinical application, we developed a lipid nanoparticle (LNP) system designed to deliver siPDLIM4, DDP, or siPDLIM4/DDP specifically to GC tissues. Our research demonstrated that LNPs containing either siPDLIM4 or DDP exhibit anti-tumor effects on GC, however, the combined siPDLIM4/DDP LNPs show the most pronounced anti-tumor efficacy, providing a novel perspective for therapeutic strategies against this disease.

Literatures have already reported the roles of PDLIM4 and is considered to prevent tumor development in several cancers. The investigation revealed that prostate cancer cells display reduced protein and mRNA levels of PDLIM4 [41]. Additionally, PDLIM4 overexpression hinders ovarian cancer cell growth and invasion by blocking the STAT3 signaling pathway [10]. However, there is a lack of research elucidating the role of PDLIM4 in GC and its sensitivity to DDP. In this study, a comprehensive bioinformatics analysis was conducted, leading to the identification of PDLIM4 as a significant factor. The bioinformatics analysis revealed that GC patients exhibiting elevated PDLIM4 expression experienced a poorer prognosis. Furthermore, PDLIM4 was notably overexpressed in GC tissues. Subsequently, our research confirmed that PDLIM4 promote the malignant progression of GC cells.

To elucidate the possibility mechanism of PDLIM4 in GC cells, HEK-293 T cells was utilized to identify 94 proteins interacting with PDLIM4 through co-immunoprecipitation and mass spectrometry techniques. These identified proteins were subsequently intersected with those predicted by BioGRAD, revealing HSP70 and HSC70 as the most likely interactors with PDLIM4. Analysis of clinical data further identified HSP70 as an interacting gene of PDLIM4. Moreover, we confirmed that increased expression of PDLIM4 correlated with elevated HSP70 expression in clinical cohorts and patients exhibiting high expression levels of both PDLIM4 and HSP70 were found to have reduced OS in contrast with those with low protein expression, who demonstrated the most favorable prognosis. Our study also revealed that PDLIM4 interacts with the C-terminus of HSP70 through its own C-terminus and intermediate domains. Previous studies have demonstrated that the PDLIM family interacts with various proteins via its PDZ domain, including SNX17 [42], α-actin [43], β-catenin [44], and YAP [45]. Although the LIM domain within the PDLIM family is well recognized for its role in facilitating protein interactions and regulating transcriptional activity, there is limited evidence supporting interactions between individual LIM domains and other proteins. Furthermore, existing research has not identified protein-binding capabilities within the intermediate region of the PDLIM family. Nonetheless, our experimental findings revealed that both the LIM domain and the intermediate region of PDLIM4 can bind to HSP70. This novel discovery supplied a foundation for further exploration into the intermediate region of the PDLIM family.

Currently, there is a lack of definitive literature documenting a direct interaction between PDLIM4 and HSP70. Nevertheless, our research team has identified and confirmed this interaction for the first time. Our experimental findings indicate that PDLIM4 modulates the stability of HSP70 through the ubiquitin–proteasome pathway. Specifically, PDLIM4 binds to the C-terminus of HSP70, thereby influencing its ubiquitination and subsequent degradation. Given that neither PDLIM4 nor HSP70 are classified as ubiquitin-related genes, we hypothesized the involvement of an E3 ubiquitin ligase or deubiquitinase that may function either synergistically or competitively. Through a comprehensive review of the literature, we identified STUB1 [46–48] as an E3 ubiquitin ligase that interacts with the C-terminus of HSP70 and regulates its ubiquitination [49–51]. In neurodegenerative diseases, the interaction between STUB1 and HSP70 is acknowledged as a pivotal regulatory mechanism [52]. In light of this, we undertook comprehensive research on STUB1. Our experimental findings reveal that the PDLIM4 mediated ubiquitination and degradation of HSP70 are contingent upon STUB1. Mechanistically, PDLIM4 competes with STUB1 for binding to HSP70, thereby inhibiting the interaction between STUB1 and HSP70. This interference subsequently impedes the STUB1 mediated ubiquitination and degradation of HSP70. This study is the first to propose a competitive interaction between PDLIM4 and STUB1 for HSP70, thereby offering a novel contribution to the field.

DDP is extensively utilized as a chemotherapeutic agent in the treatment of GC, however, its therapeutic efficacy is significantly undermined by the development of resistance. CircAKT3 promotes the expression of PIK3R1 by suppressing miR-198, thereby increasing DDP resistance in GC cells [53]. Furthermore, RECQL4 has been implicated in facilitating DDP resistance in GC [54]. In summary, DDP resistance in GC treatment is characterized by multiple complex molecular mechanisms. In our study, the findings suggest that PDLIM4 is critically involved in the malignant progression of GC and in modulating the sensitivity of GC cells to DDP. Experiments conducted both in vivo and in vitro provided evidence that PDLIM4 KD enhances the sensitivity of GC cells to DDP in a manner dependent on HSP70. Notably, HSP70 is significantly overexpressed in various cancers [55, 56] and is closely associated with resistance to chemotherapy [57, 58]. Previous studies have shown that HSP70 mitigates DDP induced apoptosis in HGC-27 cells by modulating the MAPK signaling pathway [19]. Additionally, in osteosarcoma, HSP70 establishes a feedback loop through the JNK/JUN signaling pathway, thereby influencing cellular resistance to DDP [59]. Collectively, these findings highlight the significance of HSP70 in regulating sensitivity to DDP via the MAPK pathway. Consequently, we hypothesize that PDLIM4 influences the MAPK pathway by enhancing the stability of HSP70, thereby contributing to the malignant progression and DDP resistance of GC. This study identifies the PDLIM4-HSP70-MAPK axis as a prospective therapeutic target for GC and provides a theoretical framework for future research.

Recent advancements in nanotechnology have significantly improved cancer treatment strategies, particularly in the context of co delivering siRNA and chemotherapeutic agents [60–62]. Lipid nanoparticles (LNPs) have emerged as a highly sophisticated platform for nucleic acid delivery, attracting substantial interest due to their compact structure and effectiveness in encapsulating and delivering siRNA. In this study, we developed siPDLIM4 LNPs, DDP LNPs, and siPDLIM4/DDP LNPs for the treatment of GC. Our results indicate that siPDLIM4/DDP LNPs provide effective protection for siPDLIM4 and DDP against premature degradation, thereby enhancing cellular uptake efficiency and facilitating the successful delivery of siPDLIM4 and DDP to tumor cells. In vivo studies have notably demonstrated that tumor cells show an increased uptake of LNPs, potentially accelerating their accumulation within tumor sites. Functional assays have indicated that both siPDLIM4 LNPs and DDP LNPs exert inhibitory effects on tumor proliferation when compared to LNPs. Furthermore, the siPDLIM4/DDP LNP significantly suppresses tumor growth. HE staining along with blood biochemistry analyses, indicated that none of the LNP groups induce significant toxicity. Consequently, the combination of siRNA and DDP nanoparticles holds promise for enhancing the efficacy and safety of GC treatment in future research. In recent advancements within nanoparticle research, considerable attention has been directed towards the clinical translational potential of lipid nanoparticles (LNPs) encapsulating small interfering RNA (siRNA) and chemotherapeutic agents [29, 62]. The siPDLIM4/DDP LNPs represent a synergistic integration of the gene silencing capabilities of siRNA with the cytotoxic properties of chemotherapeutic drugs, aiming to enhance the precision and efficacy of cancer therapies. Notably, the siPDLIM4/DDP encapsulated by lipid nanoparticles demonstrates significant therapeutic effects in gastric cancer (GC) patients exhibiting high expression levels of PDLIM4, and it has the potential to mitigate cisplatin resistance in these patients. This strategy has the potential to not only enhance treatment efficacy but also to mitigate drug side effects and overcome drug tolerance, warranting further research and development. However, significant challenges remain in the clinical translation process, particularly concerning the delivery efficiency of siRNA, biocompatibility, and potential immune responses [63–65]. Future research should focus on improving the targeting and delivery efficiency of nanoparticles while reducing their immunogenicity and toxicity. The integration of artificial intelligence and machine learning techniques offers promising avenues for optimizing nanoparticle design, thereby enabling more effective co-delivery of siRNA and therapeutic agents [66, 67]. Furthermore, the innovation of novel biologically responsive nanomaterials could enable controlled drug release in response to specific physiological conditions, thereby enhancing the precision and safety of therapeutic interventions [68, 69].

## Conclusions

In this study, we found that PDLIM4 expression was notably higher in GC tissues and was positive linked to GC stages, poor prognosis, and DDP resistance. Mechanistically, PDLIM4 was found to competitively bind to HSP70 in conjunction with STUB1, thereby inhibiting the ubiquitination and subsequent degradation of HSP70, which ultimately resulted in the activation of the MAPK signaling pathway. Notably, our team have developed lipid nanoparticles (LNPs) that effectively encapsulate siPDLIM4 and DDP, demonstrating anti-tumor efficacy both in vivo and in vitro (Graphical Abstract). Consequently, targeting the PDLIM4-HSP70-MAPK axis could potentially represent a new therapeutic approach for GC treatment.

## Supplementary Information

Additional file1

Additional file2

Additional file3

Additional file4

## Data Availability

No datasets were generated or analysed during the current study.

## Abbreviations

GC: Gastric cancer
PDLIM4: PDZ and LIM domain protein 4
HSP70: Heat Shock Protein 70
STUB1: STIP1 homology and U-box containing protein 1
LNPs: Lipid nanoparticles
IARC: International Agency for Research on Cancer
RhoGDI2: Rho GDP dissociation inhibitor 2
DDP: Cisplatin
IHC: Immunohistochemistry
IF: Immunofluorescence
CO-IP: Co-immunoprecipitation
ALT: Alanine aminotransferase
AST: Aspartate aminotransferase
Crea: Creatinine
TCGA: The cancer genome atlas
GEO: Gene expression omnibus
GDSC: Genomics of drug sensitivity in cancer
KM: Kaplan–meier
TPM: Transcripts per million
OS: Overall survival
PFI: Progression-free interval
DSS: Disease-specific survival
Co-IP: Co-immunoprecipitation
CHX: Cycloheximide
TEM: Transmission electron microscopy
BioD: Biodistribution
PK: Pharmacokinetics
PLA: Proximity ligation assay
