# Correction: PDLIM4 drives gastric cancer malignant progression and cisplatin resistance by inhibiting HSP70 ubiquitination and degradation via competitive interaction with STUB1

**DOI:** 10.1186/s12951-026-04118-6

**Published:** 2026-02-26

**Authors:** Chao Zhu, Meng Chen, Linwei Fan, Yu Wang, Mengwei Liu, Guiyu Kang, Fang Yin, Hong Tang, Yun He, Sifan Zhang, Linda Zeng, Wei Liu, Kuai Yu, Aiping Le

**Affiliations:** 1 Department of Transfusion Medicine, Key Laboratory of Jiangxi Province for Transfusion Medicine, The First Affiliated Hospital, Jiangxi Medical College, Nanchang University, Nanchang, 330006 Jiangxi China; 2 Postdoctoral Research Station, The First Affiliated Hospital, Jiangxi Medical College, Nanchang University, Nanchang, 330006 People’s Republic of China; 3 Department of Hematology, The First Affiliated Hospital of Nanchang University, Nanchang, 330006 China

**Correction: Journal of Nanobiotechnology (2025) 23:661**

10.1186/s12951-025-03720-4

Unfortunately, in Fig. 10h, the unit for Crea was erroneously indicated as "mmol/L" instead of the correct unit, "μmol/L."

For completeness and transparency, the correct and incorrect versions of Fig. 10 is displayed below.

**Incorrect Fig. 10**

**Fig. 10:**
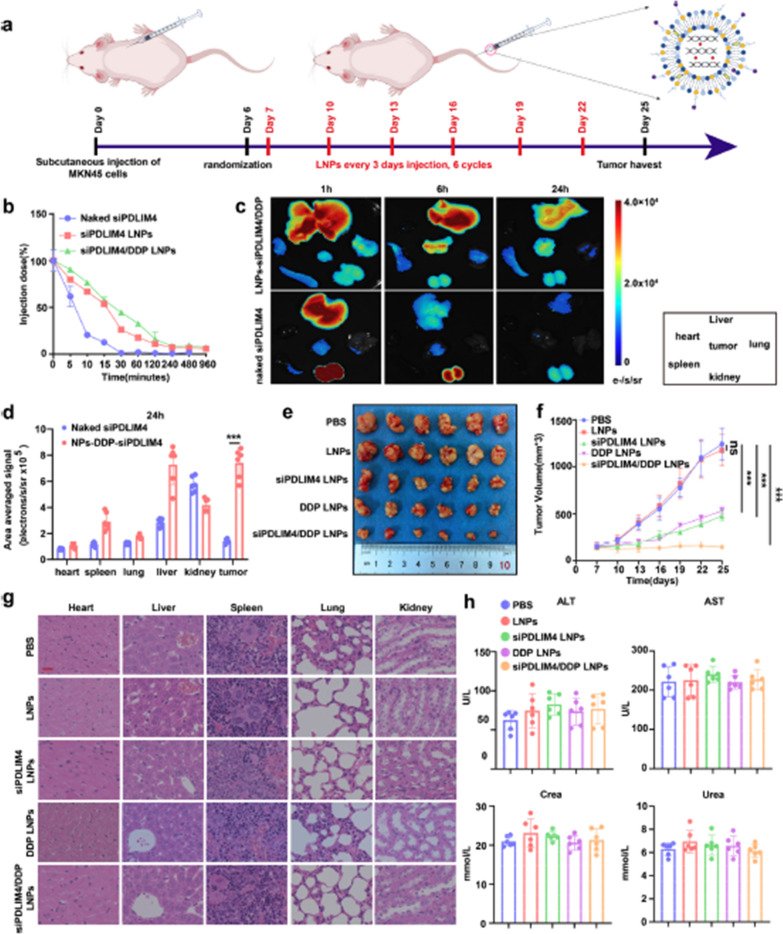
In vivo evaluation of the therapeutic efficacy and toxicity of siPDLIM4/DDP LNPs. **a** A schematic diagram illustrates the timeline for tumor implantationand the administration of PBS, LNPs, siPDLIM4 LNPs, DDP LNPs, and siPDLIM4/DDP LNPs in mice bearing MKN45 tumors. The mice receivedLNPs injections at three-day intervals for a total of six cycles. **b** The pharmacokinetics of naked siPDLIM4, siPDLIM4 LNPs and siPDLIM4/DDP LNPs wereevaluated in vivo. **c** Naked CY5-siPDLIM4 and CY5-siPDLIM4/DDP LNPs were administered intravenously and 1, 6, 24 hours post-injection, tumors andmajor organs were harvested for biodistribution analysis using small animal CT/live imaging all-in-one machine (Milabs B.V.). **d** Quantitative data revealedthe distribution of naked siPDLIM4 and siPDLIM4/DDP LNPs across various organs, including tumors, in MKN45 tumor bearing mice after 24 hours postinjection. **e **Representative images of tumors (n = 6) are provided. **f** The progression of tumor volume in vivo is depicted. **g** Hematoxylin and eosin (H&E)stained images of key organs are presented following treatment with PBS, LNPs, siPDLIM4 LNPs, DDP LNPs, and siPDLIM4/DDP LNPs, scale bar: 20 μm. **h** Serum levels of ALT, AST, creatinine, and urea were measured following treatment with PBS, LNPs, siPDLIM4 LNPs, DDP LNPs, and siPDLIM4/DDP LNPs. Datawere presented as means ± SD. ***P<0.001; ns, no statistical difference

**Correct Fig. 10**

**Fig. 10:**
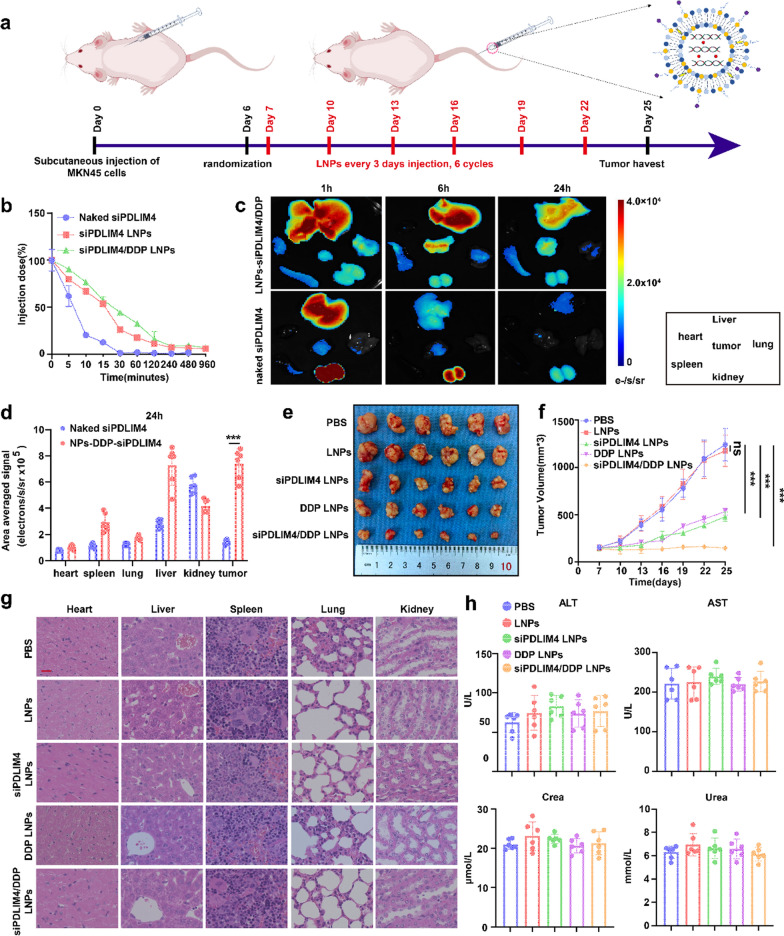
In vivo evaluation of the therapeutic efficacy and toxicity of siPDLIM4/DDP LNPs. **a** A schematic diagram illustrates the timeline for tumor implantation and the administration of PBS, LNPs, siPDLIM4 LNPs, DDP LNPs, and siPDLIM4/DDP LNPs in mice bearing MKN45 tumors. The mice received LNPs injections at three-day intervals for a total of six cycles. **b** The pharmacokinetics of naked siPDLIM4, siPDLIM4 LNPs and siPDLIM4/DDP LNPs were evaluated in vivo. **c** Naked CY5-siPDLIM4 and CY5-siPDLIM4/DDP LNPs were administered intravenously and 1, 6, 24 hours post-injection, tumors and major organs were harvested for biodistribution analysis using small animal CT/live imaging all-in-one machine (Milabs B.V.). **d** Quantitative data revealed the distribution of naked siPDLIM4 and siPDLIM4/DDP LNPs across various organs, including tumors, in MKN45 tumor bearing mice after 24 hours postinjection. **e** Representative images of tumors (n = 6) are provided. **f** The progression of tumor volume in vivo is depicted. **g** Hematoxylin and eosin (H&E) stained images of key organs are presented following treatment with PBS, LNPs, siPDLIM4 LNPs, DDP LNPs, and siPDLIM4/DDP LNPs, scale bar: 20 μm. **h** Serum levels of ALT, AST, creatinine, and urea were measured following treatment with PBS, LNPs, siPDLIM4 LNPs, DDP LNPs, and siPDLIM4/DDP LNPs. Datawere presented as means ± SD. ***P<0.001; ns, no statistical difference

