# Supplementary material for: PDLIM4 drives gastric cancer malignant progression and cisplatin resistance by inhibiting HSP70 ubiquitination and degradation via competitive interaction with STUB1
Source: J Nanobiotechnology. 2025 Oct 11;23:661. doi: 10.1186/s12951-025-03720-4 (PMC12514821; doi:10.1186/s12951-025-03720-4)
Supplement: Supplementary file 1 — Additional file1 [file 12951_2025_3720_MOESM1_ESM.docx]

**Cell Lines and Cell Culture**

MKN45, HGC27 and HEK-293T cells were preserved under standard laboratory conditions. HEK-293T cells utilized DMEM (GIBCO, USA) for cultivation, whereas MKN45 and HGC27 cells all were cultured in RPMI 1640 medium (GIBCO, USA). In a humidified atmosphere containing 5% CO2, the cells were incubated at 37°C with culture media that was supplemented with 10% fetal bovine serum (FBS) (Cellmax, China) and 1% penicillin/streptomycin.

**Quantitative Real-Time PCR (RT-qPCR)**

The standard Trizol protocol was utilized to obtain total RNA from cultured cells and GC tissues. Subsequently, cDNA was synthesized using the Transcript® II All-in-One First-Strand cDNA Synthesis SuperMix for qPCR kit (TransGen Biotech, China). RT-qPCR was then conducted utilizing the ChamQ Universal SYBR qPCR Master Mix (Vazyme, China). Data analysis was performed using the 2^-ΔΔCT^ method, and the results are shown as relative expression levels, β-ACTIN served as the internal control. Primer sequences for RT-qPCR are listed in **Table S1**.

**Cell proliferation and migration assay**

To assess cell proliferation capacity, we employed colony formation assays, cell counting assays, and CCK-8 assays. In the colony formation assays, GC cells were placed into 6-well plates at a concentration of 1×10^3 cells per well and grown for a period of 7 to 14 days. The procedure involved fixing cells with 4% paraformaldehyde for 30 minutes and staining them with 0.1% crystal violet for 15 minutes. In the cell counting experiments, GC cells were seeded into 12-well plates at a concentration of 1×10^4 cells per well, and their proliferation was monitored every two days until 6th day. In the CCK-8 experiments, 2,000 cells were placed into 96-well plates and observed at 0, 24, 48, 72, and 96 hours. The CCK-8 reagent (MedChemExpress, China) was added at each time point and incubated for 2 hours, after which the absorbance at 450 nm was measured with a microplate reader. During the Transwell migration assay, 600 µL of medium with 20% serum was added to the lower chamber and a total of 3×10^4 GC cells were added to the upper chamber in 200 µL of medium lacking serum. Cells that traveled to the membrane's lower side, after being treated with 4% paraformaldehyde, the samples were stained with crystal violet and imaged using a microscope.

**Cell survival assay**

Initially, in a 96-well plate, 5,000 GC cells were seeded and allowed to adhere before being exposed to pharmacological treatments for 24 hours. Once the incubation period was over, each well received the CCK-8 reagent and was incubated for addition 2 hours. The measurement of absorbance at 450 nm with a microplate reader was used to assess cell viability.

**Cell apoptosis analysis**

The assessment of apoptosis was performed using either an Annexin V-APC/PI kit or an Annexin V-FITC/PI kit. 500,000 cells were placed in a 6-well plate and incubated for a day. Cells were collected, resuspended in buffer, stained with FITC or APC for 10 minutes, and then stained with PI for 5 minutes prior to flow cytometry analysis.

**Identification of Prognosis-Related Genes in Gastric Cancer**

Initially, univariate Cox regression and Kaplan-Meier (KM) analyses were conducted on the GSE66229 dataset and the TCGA cohort, which utilizing the survival R package. This approach facilitated the identification of differentially expressed genes common to both cohorts, thereby enabling the determination of genes associated with OS in GC patients. Genes exhibiting P values ≤ 0.05 in both analyses were selected for further investigation. Subsequently, the glmnet R package was employed to refine the selection of prognostic genes through the application of least absolute shrinkage and selection operator (Lasso) regression.

**Drug Sensitivity Analysis**

Drug sensitivity analysis was conducted utilizing the oncoPredict R package, employing the half-maximal inhibitory concentration (IC50) as the evaluation metric. Drug sensitivity data from GDSC2 within the GDSC database, alongside expression data from the GEO database (datasets GSE122401), were normalized to transcripts per million (TPM) formats. The GDSC2 data functioned as the training set, whereas the GEO datasets were designated as test sets. This analysis assessed the influence of PDLIM4 expression levels on drug sensitivity and their correlations, with results visualized using the R packages ggplot2, ggpubr, tidyverse, magrittr, and ggstatsplot.

**Stability assay**

The siPDLIM4/DDP LNPs were subjected to incubation at 37°C in PBS with 10% FBS or PBS (0.01 M, pH 7.4). The dynamic light scattering (DLS) technique was subsequently applied to monitor the nanoparticle size at predetermined time intervals.

**Cellular Uptake**

GC cells were inoculated into a confocal dish. After cell adhesion, the cells were treated with naked siPDLIM4 and siPDLIM4/DDP LNPs for four hours. Subsequently, the cells were incubated with Lyso-Tracker Green (Beyotime, China) for one hour. Hoechst 33342 was used for nuclear staining for ten minutes.To capture the images, a confocal laser scanning microscope (ZEISS, Germany) was used.

**In vitro Transfection Efficiency**

GC cells were placed into six-well plate and subsequently exposed to PBS, 1640 culture medium, LNPs, naked siPDLIM4, and siPDLIM4/DDP LNPs for 24 hours. The fluorescence expression of CY5 was analyzed using flow cytometry.
