# Supplementary material for: PDLIM4 drives gastric cancer malignant progression and cisplatin resistance by inhibiting HSP70 ubiquitination and degradation via competitive interaction with STUB1
Source: J Nanobiotechnology. 2025 Oct 11;23:661. doi: 10.1186/s12951-025-03720-4 (PMC12514821; doi:10.1186/s12951-025-03720-4)
Supplement: Supplementary file 2 — Additional file2 [file 12951_2025_3720_MOESM2_ESM.docx]

Supplementary Materials for

**PDLIM4 Drives Gastric Cancer Malignant Progression and** **Cisplatin Resistance by Inhibiting HSP70 Ubiquitination and Degradation via Competitive Interaction with STUB1.**

Chao Zhu^1^**^†^**, Meng Chen^2,3^**^†^**, Linwei Fan^1^**^†^**, Yu Wang^1^, Mengwei Liu^1^, Guiyu Kang^1^, Fang Yin^1^, Hong Tang^1^, Yun He^1^, Sifan Zhang^1^, Linda Zeng^1^, Wei Liu^1^*, Kuai Yu^1^*, Aiping Le^1^*

Correspondence to: [ndyfy00973@ncu.edu.cn](mailto:ndyfy00973@ncu.edu.cn)

**This PDF file includes:**

Figures. S1 to S10

Tables. S1

**
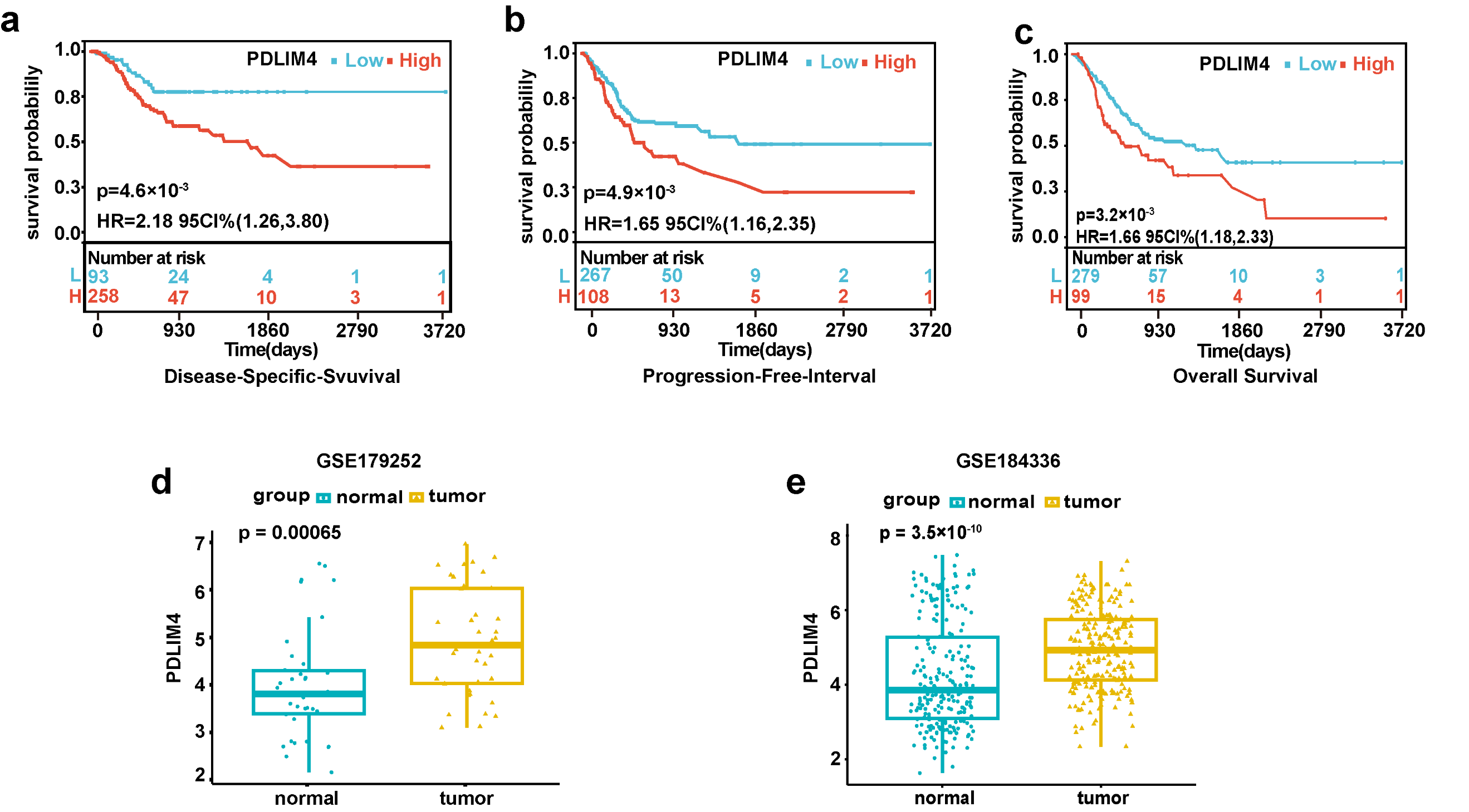
**

**Figure S1 a-c** Disease-specific survival (DSS), progression-free interval (PFI), and overall survival (OS) were analyzed according to PDLIM4 expression levels within the TCGA dataset. **d-e** Variations in PDLIM4 expression between GC and normal tissues were analyzed using the GSE179252 and GSE184336 datasets.


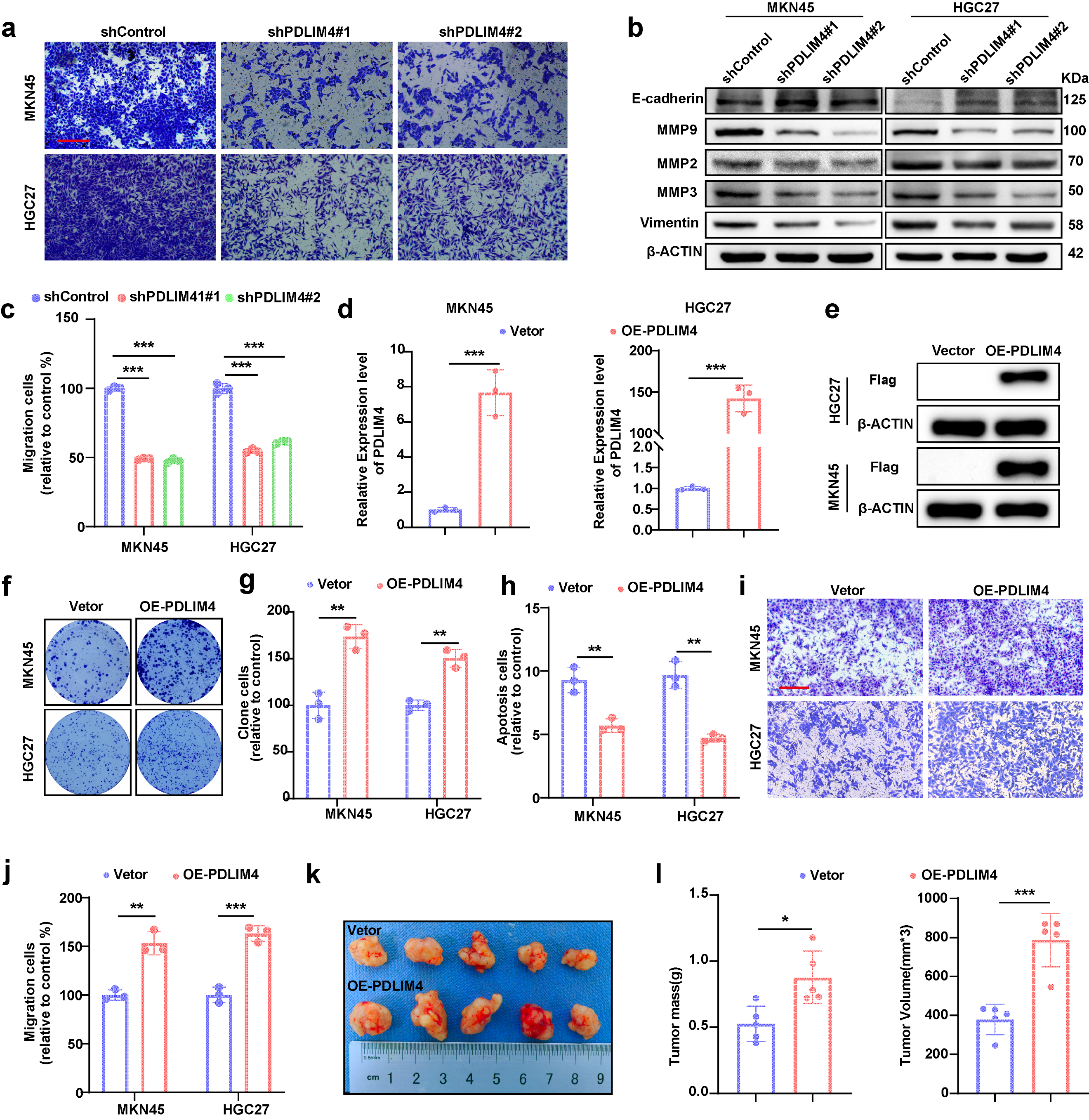


**Figure S2 a, c** Cell migration of MKN45 and HGC27 cells with the depletion of PDLIM4, scale bars: 200μm. **b** western blotting detects transfer related indicators of MKN45 and HGC27 cells with the depletion of PDLIM4. **d-e** RT-qPCR and western blotting experiments were used to detect the overexpression efficiency of PDLIM4 in MKN45 and HGC27 cells. **f-g** Clones formation in MKN45 and HGC27 cells with overexpression of PDLIM4. **h** Apoptosis of MKN45 and HGC27 cells with the overexpression of PDLIM4. **i-j** Cell migration of MKN45 and HGC27 cells with the overexpression of PDLIM4, scale bars: 200μm. **k** Representative images of tumors harvested from mice containing vector or OE-PDLIM4 MKN45 cells. **l** Weight and volume of the harvested tumors (n = 5). Data were presented as means±SD. *P<0.05; **P<0.01; ***P<0.001.


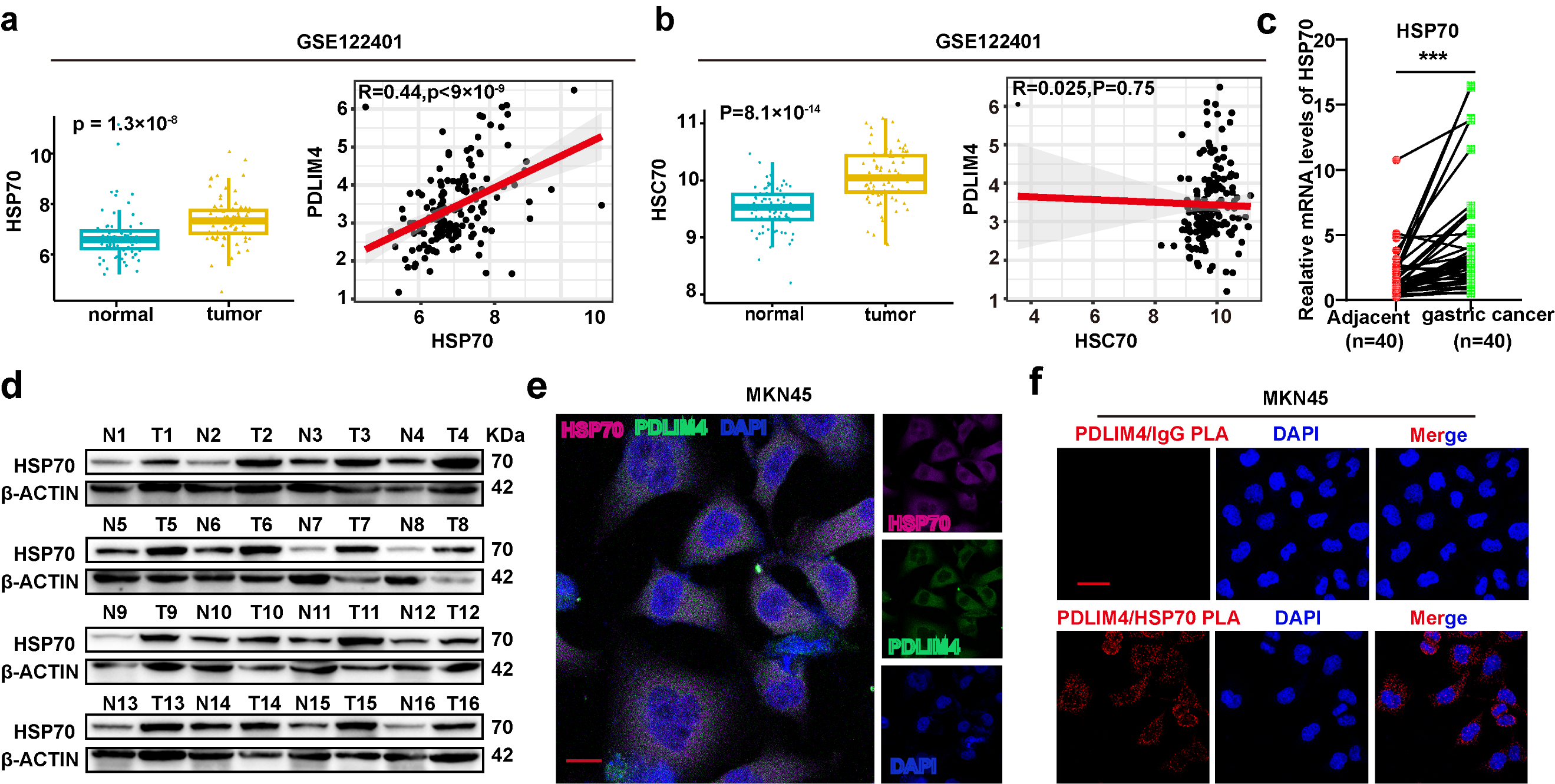


**Figure S3 a** Within GSE122401, the mRNA expression of HSP70 was measured in GC and normal tissues, and the relationship between PDLIM4 and HSP70 in GC tissues was assessed using Spearman's correlation. **b** Within GSE122401, the mRNA expression of HSC70 was measured in GC and normal tissues, and the relationship between PDLIM4 and HSC70 in GC tissues was assessed using Spearman's correlation. **c** The levels of HSP70 mRNA in 40 pairs of GC tissues and adjacent normal tissues. **d** Representative western blotting results showing the HSP70 protein levels in 16 pairs of GC tissues and adjacent normal tissues. **e** Immunofluorescence staining of PDLIM4 and HSP70 in MKN45 cell, Scale bar: 20μm. f The interaction between PDLIM4 and HSP70 in MKN45 cells was detected by PLA, Scale bar: 20μm. Data were presented as means±SD. ***P<0.001.

**
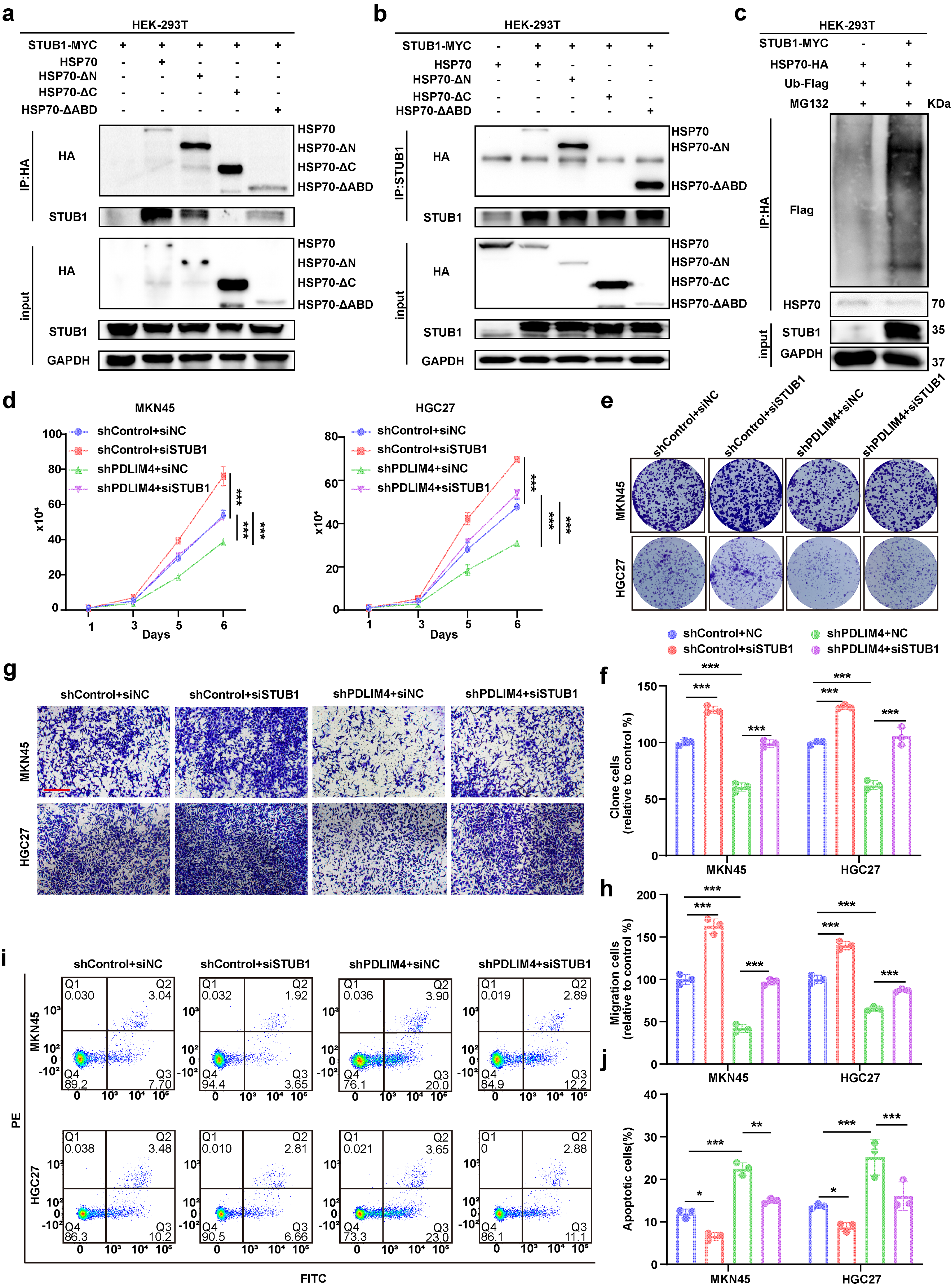
**

**Figure S5 a-b** Interaction between exogenous HSP70, HSP70-ΔN, HSP70-ΔC, HSP70-ΔABD and STUB1 in HEK-293T cells by CO-IP assay. **c** HSP70 ubiquitination levels in HEK-293T cells with STUB1 overexpression. **d** Cell growth ability of MKN45 and HGC27 cells transfected with shControl + siNC, shControl + siSTUB1, shPDLIM4 + siNC, and shPDLIM4+ siSTUB1. **e-f** Colony formation ability of MKN45 and HGC27 cells transfected with shControl + siNC, shControl + siSTUB1, shPDLIM4 + siNC, and shPDLIM4+ siSTUB1. **g-h** Cell migration ability of MKN45 and HGC27 cells transfected with shControl + siNC, shControl + siSTUB1, shPDLIM4 + siNC, and shPDLIM4+ siSTUB1, scale bars: 200μm. **i-j** Apoptosis ability of MKN45 and HGC27 cells transfected with shControl + siNC, shControl + siSTUB1, shPDLIM4 + siNC, and shPDLIM4+ siSTUB1. Data were presented as means±SD. *P<0.05; **P<0.01; ***P<0.001.


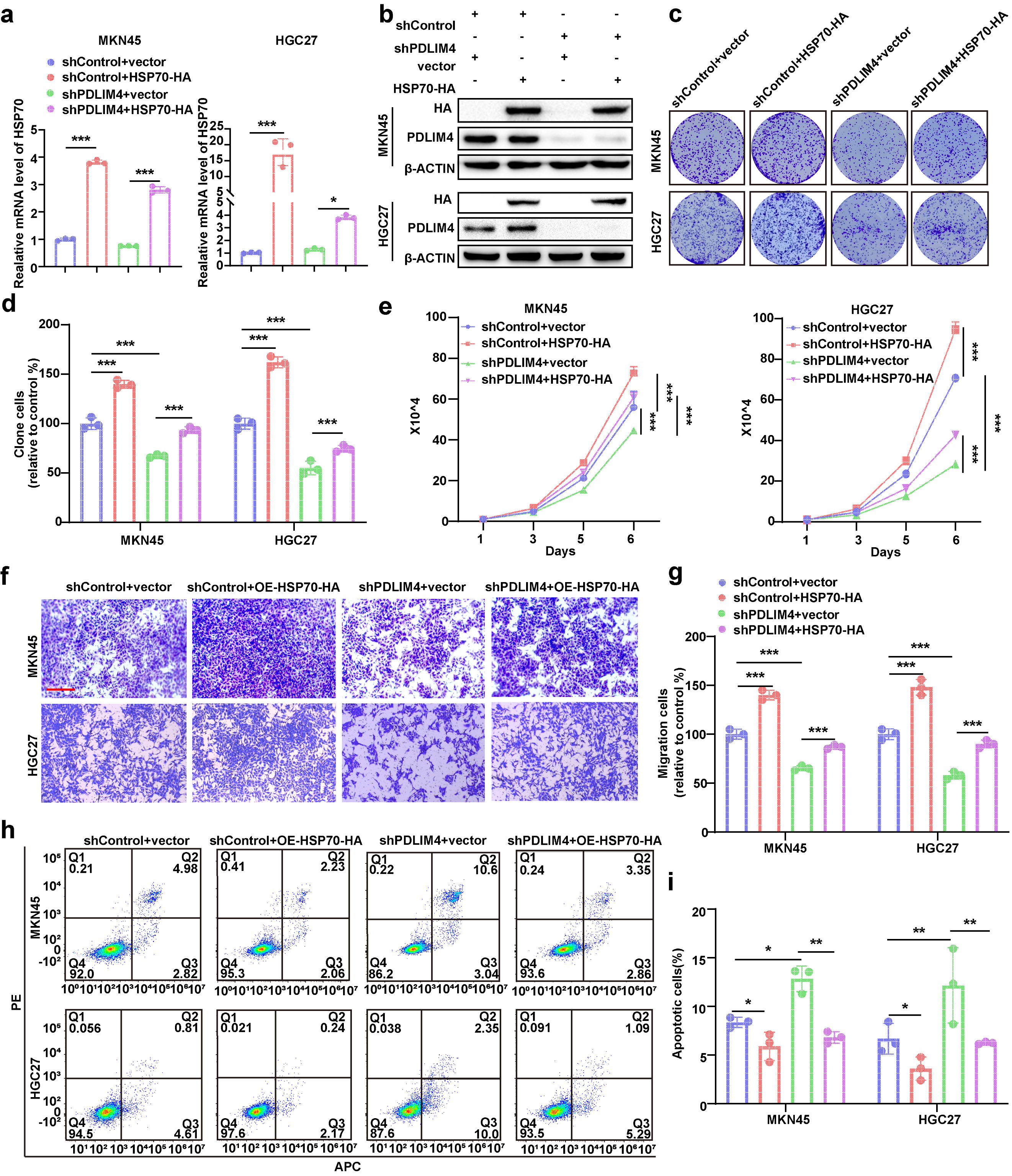


**Figure S6 a** RT-qPCR was used to assess the overexpression efficiency of HSP70 at mRNA level in PDLIM4 KD MKN45 and HGC27 cells. **b** Western blotting was used to analysis the overexpression efficiency of HSP70 at protein level in PDLIM4 KD MKN45 and HGC27 cells. **c-d** Colony formation ability of MKN45 and HGC27 cells transfected with shControl + vector, shControl + HSP70-HA, shPDLIM4 + vector, and shPDLIM4+ HSP70-HA. **e** Cell growth ability of MKN45 and HGC27 cells transfected with shControl + vector, shControl + HSP70-HA, shPDLIM4 + vector, and shPDLIM4+ HSP70-HA. **f-g** Cell migration ability of MKN45 and HGC27 cells transfected with shControl + vector, shControl + HSP70-HA, shPDLIM4 + vector, and shPDLIM4+ HSP70-HA, scale bars: 200μm. **h-i** Apoptosis ability of MKN45 and HGC27 cells transfected with shControl + vector, shControl + HSP70-HA, shPDLIM4 + vector, and shPDLIM4+ HSP70-HA. Data were presented as means±SD. *P<0.05; **P<0.01; ***P<0.001.


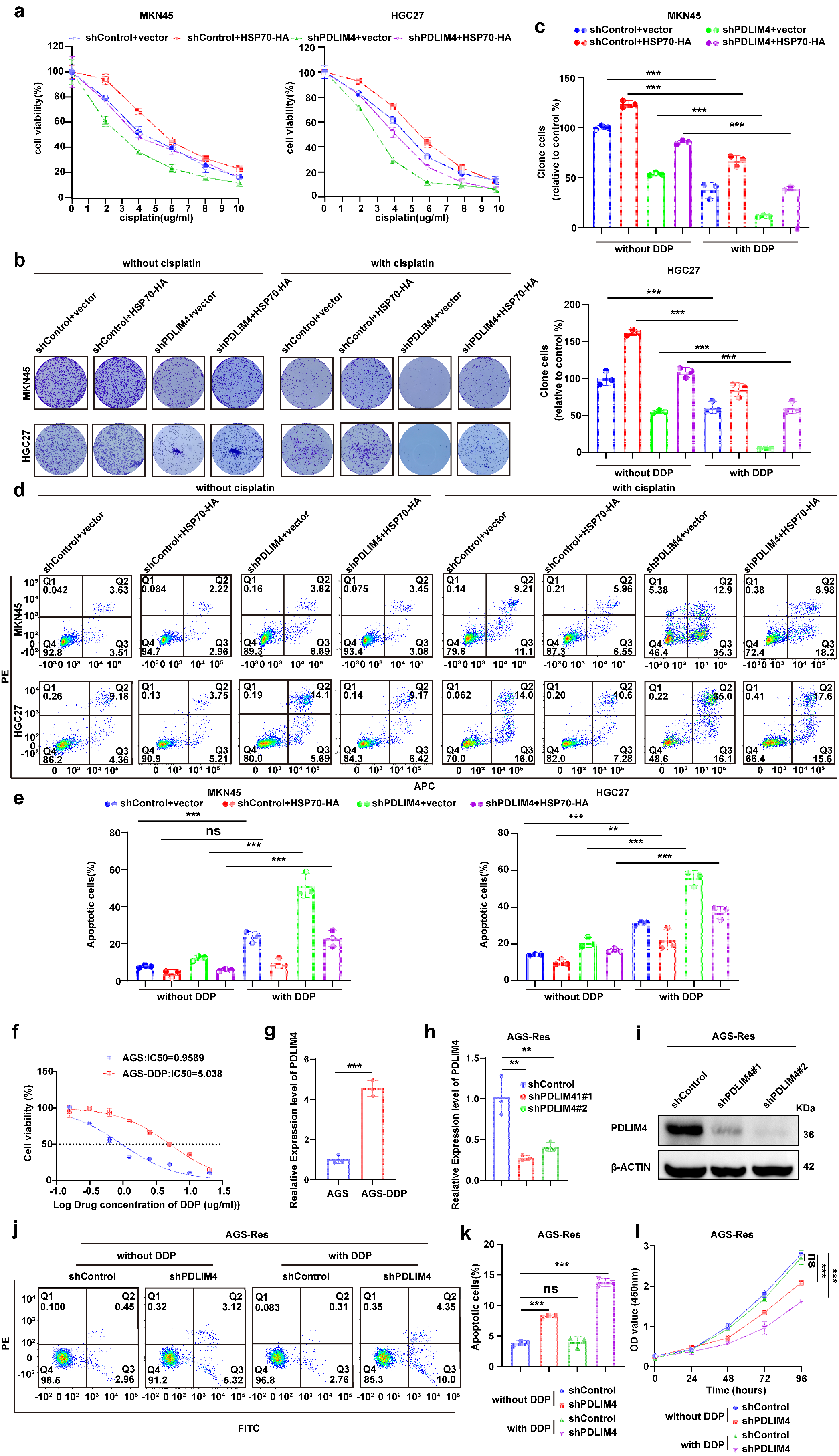


**Figure S7 a** Assessment of cell viability in PDLIM4 KD cells with overexpression of HSP70 following a 24-hour treatment with DDP. **b-c** Evaluation of colony formation capacity in PDLIM4 KD cells with overexpression of HSP70 subjected to a 24-hour DDP treatment. **d-e** Examination of apoptotic activity in PDLIM4 KD cells with overexpression of HSP70 after 24 hours of DDP exposure. **f** Assessment of cell viability in AGS sensitive and AGS resistant cells following a 72-hour treatment with DDP. **g** RT-qPCR were used to detect the mRNA expression of PDLIM4 in AGS resistant and AGS sensitive cells. **h-i** RT-qPCR and western blotting experiments were used to detect the knockdown efficiency of PDLIM4 in AGS resistant cells. **j-k** Examination of apoptotic activity in AGS resistant cells with knockdown of PDLIM4 after 24 hours of DDP exposure. **l** Cell growth ability of AGS resistant cells transfected with shControl, shPDLIM4 following treatment with DDP. Data were presented as means±SD. **P<0.01; ***P<0.001; ns, no statistical difference.

**
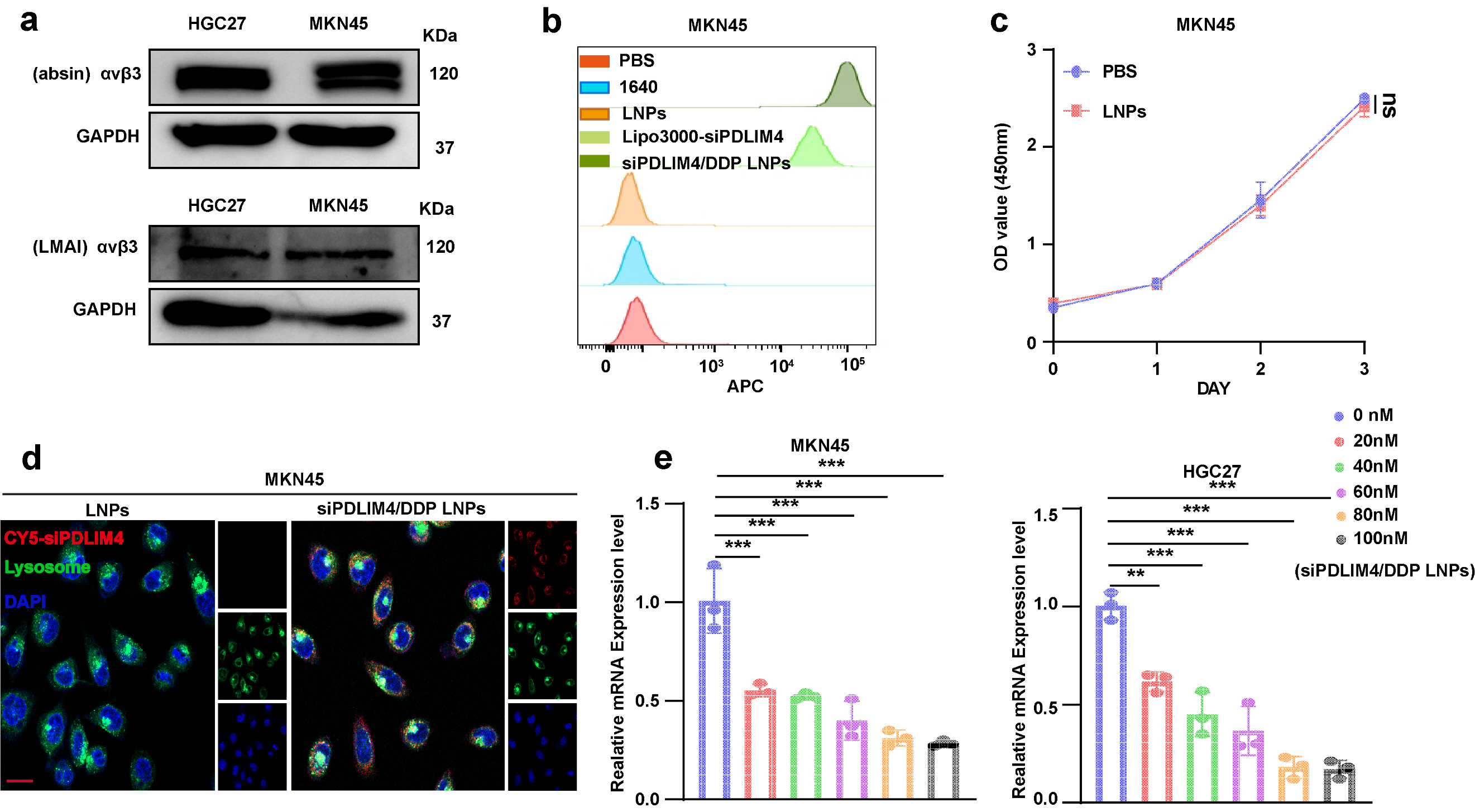
**

**Figure S8 a** Western blotting detected the protein level of αvβ3 in MKN45 and HGC27 cells using αvβ3 antibodies from two different manufacturers. **b** Proportion of CY5 positive cells in MKN45 cells were detected by flow cytometry. **c** The survival rate of MKN45 cells following exposure to PBS or LNPs. **d** After 4 hours of incubation with LNPs and siPDLIM4/DDP LNPs, confocal laser scanning microscopy images of MKN45 cells were taken. Endosomes were stained green with Lysotracker, nuclei were stained blue with Hoetest33342, and siPDLIM4 was labeled with CY5, scale bars: 20μm. **e** Detection of PDLIM4 KD efficiency in MKN45 and HGC27 cells after adding different concentrations of siPDLIM4/DDP LNPs using RT-qPCR. Data were presented as means±SD. **P<0.01; ***P<0.001; ns, no statistical difference.


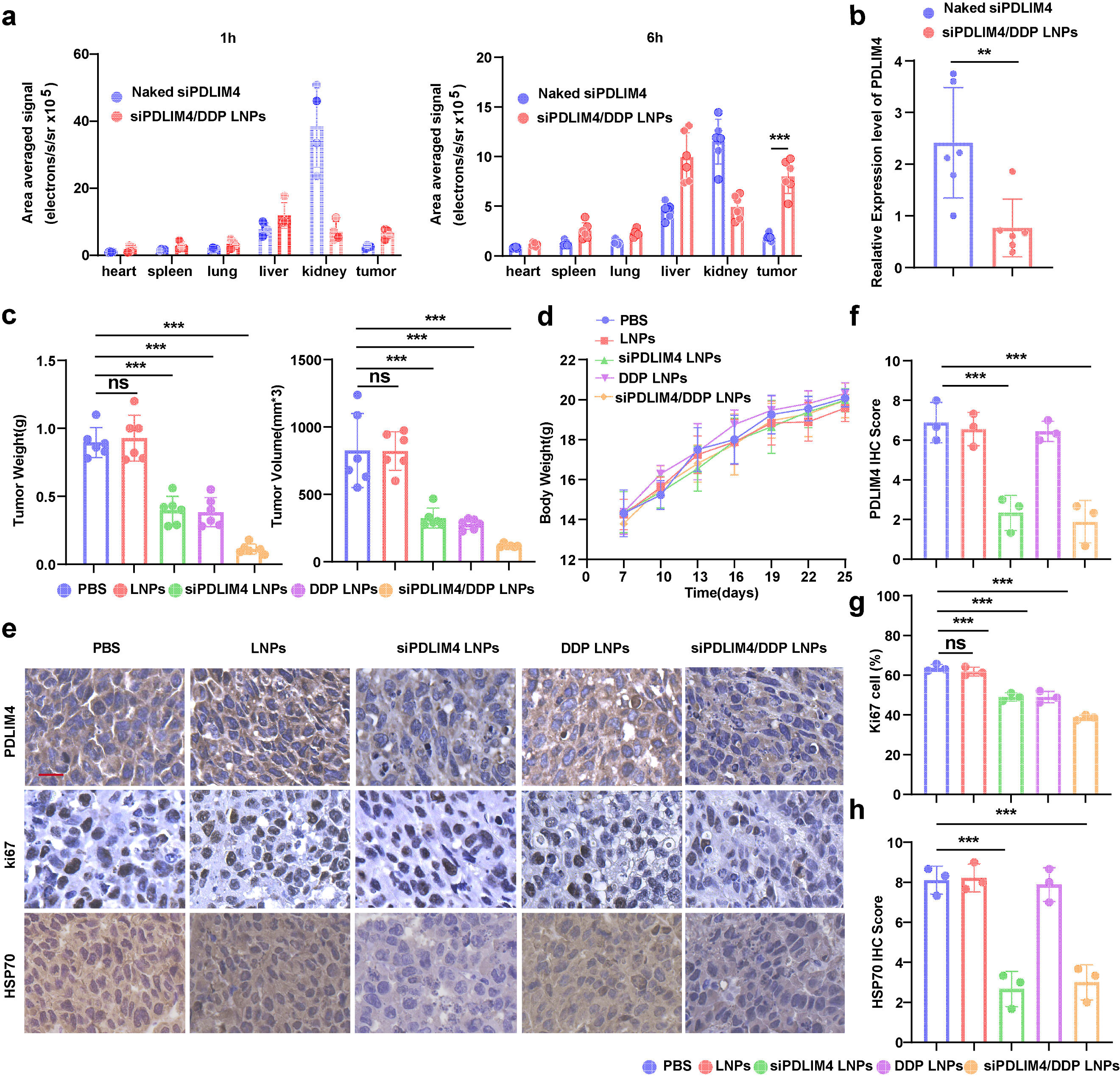


**Figure S10 a** Quantitative data revealed the distribution of naked siPDLIM4 and siPDLIM4/DDP LNPs across various organs, including tumors, in MKN45 tumor bearing mice after 1, 6 hours post-injection. **b** RT-qPCR were used to detect the expression of PDLIM4 in the tumor tissue after tail vein injection naked siPDLIM4 and CY5 siPDLIM4/DDP LNPs for 24 hours**. c** Weight and volume of the harvested tumors (n = 6). **d** Weight of the nude mice in the indicated group. **e-h** Evaluation of PDLIM4, Ki67 and HSP70 expression using IHC staining in tumor tissues after systemic treatment in several groups of mice, scale bars: 20μm. Data were presented as means±SD. **P<0.01; ***P<0.001; ns, no statistical difference.

**Table S1**

**Table S1 Primer Sequences for RT-qPCR, siRNA and shRNA**

1

| β-actin | F | 5′-CACCATTGGCAATGAGCGGTTC-3′ | RT-qPCR |
| --- | --- | --- | --- |
| PDLIM4  HSP70  STUB1 | R  F  R  F  R  F  R | 5′-AGGTCTTTGCGGATGTCCACGT-3′  5′-TGATGACAGCAAGGCTCAGGCA-3  5′-AGGCTTGGTCTGCCATCTTCTG-3  5′-ACCTTCGACGTGTCCATCCTGA-3  5′-TCCTCCACGAAGTGGTTCACCA-3  5′-TCAAGGAGCAGGGCAATCGTCT-3  5′-GCATCTTCAGGTAGCACAAGGC-3 |  |

2

| siNC  siPDLIM4  Si-STUB1 | Sense  Anti-sense  Sense  Anti-sense  Sense  Anti-sense | 5’-UUCUCCGAACGUGUCACGUTT-3′  5’-ACGUGACACGUUCGGAGAATT-3′  5’-GAACCUCAAGCAGCGUGGUUATT-3′  5’-UAACCACGCUGCUUGAGGUUCTT-3′  5’-CCCAAGUUCUGCUGUUGGACUTT-3′  5’-AGUCCAACAGCAGAACUUGGGTT-3′ | siRNA |
| --- | --- | --- | --- |

3

| ShPDLIM4#1  ShPDLIM4#2 | Sense  Anti-sense  Sense  Anti-sense | 5’-GATCGCACACAGGATCCACATCGATCTCGAGATCGATGTGGATCCTGTGTGCTTTTTG-3′  5’-AATTCAAAAAGCACACAGGATCCACATCGATCTCGAGATCGATGTGGATCCTGTGTGC-3′  5’-GATCGAACCTCAAGCAGCGTGGTTACTCGAGTAACCACGCTGCTTGAGGTTCTTTTTTG-3′  5’-AATTCAAAAAAGAACCTCAAGCAGCGTGGTTACTCGAGTAACCACGCTGCTTGAGGTTC-3′ | shRNA |
| --- | --- | --- | --- |

**4**

**SFB sequences:**

GAATTGATGGAGAAAGAAACCGCTGCTGCTAAATTCGAACGCCAGCACATGGACAGCGGAGCCGGCGCAGGAGCCGGCGCTGACGCGCCTGACTACAAAGACGATGACGACAAGGGAGATTACAAGGATGACGATGACAAAGGCGCGTCGATGGACGAGAAGACCACCGGCTGGCGGGGCGGCCACGTGGTGGAGGGCCTGGCCGGCGAGCTGGAGCAGCTGCGGGCCAGGCTGGAGCACCACCCTCAGGGCCAGCGGGAGCCCGAATTCCTCGAGAGCGGCCGCGGATCC
